# Platinum(IV) Complexes of *trans*-1,2-diamino-4-cyclohexene: Prodrugs Affording an Oxaliplatin Analogue that Overcomes Cancer Resistance

**DOI:** 10.3390/ijms21072325

**Published:** 2020-03-27

**Authors:** Paride Papadia, Katia Micoli, Alessandra Barbanente, Nicoletta Ditaranto, James D. Hoeschele, Giovanni Natile, Cristina Marzano, Valentina Gandin, Nicola Margiotta

**Affiliations:** 1 Department of Biological and Environmental Sciences and Technologies (DiSTeBA), University of Salento, 73100 Lecce, Italy; 2 Dipartimento di Chimica, Università degli Studi di Bari Aldo Moro, Via E. Orabona 4, 70125 Bari, Italy; 3 Department of Chemistry, Eastern Michigan University, Ypsilanti, MI 48197, USA; 4 Dipartimento di Scienze del Farmaco, Università di Padova, Via Marzolo 5, 35131 Padova, Italy

**Keywords:** cisplatin, oxaliplatin, anticancer drugs, platinum(IV) prodrugs, *trans*-1,2-diamino-4-cyclohexene

## Abstract

Six platinum(IV) compounds derived from an oxaliplatin analogue containing the unsaturated cyclic diamine *trans*-1,2-diamino-4-cyclohexene (DACHEX), in place of the 1,2-diaminocyclohexane, and a range of axial ligands, were synthesized and characterized. The derivatives with at least one axial chlorido ligand demonstrated solvent-assisted photoreduction. The electrochemical redox behavior was investigated by cyclic voltammetry; all compounds showed reduction potentials suitable for activation in vivo. X-ray photoelectron spectroscopy (XPS) data indicated an X-ray-induced surface reduction of the Pt(IV) substrates, which correlates with the reduction potentials measured by cyclic voltammetry. The cytotoxic activity was assessed in vitro on a panel of human cancer cell lines, also including oxaliplatin-resistant cancer cells, and compared with that of the reference compounds cisplatin and oxaliplatin; all IC_50_ values were remarkably lower than those elicited by cisplatin and somewhat lower than those of oxaliplatin. Compared to the other Pt(IV) compounds of the series, the bis-benzoate derivative was by far (5–8 times) the most cytotoxic showing that low reduction potential and high lipophilicity are essential for good cytotoxicity. Interestingly, all the complexes proved to be more active than cisplatin and oxaliplatin even in three-dimensional spheroids of A431 human cervical cancer cells.

## 1. Introduction

The clinical efficacy demonstrated by the three platinum-based drugs currently approved by the U.S. Food and Drug Administration (cisplatin, carboplatin, and oxaliplatin), has prompted over the years an intense research activity aiming to identify new metal-based anticancer drugs with enhanced efficacy, lower toxicity, and a broader spectrum of activity [1,2]. The third generation drug oxaliplatin has, in spite of two ammines, a chelating aliphatic diamine (1*R*,2*R*-diaminocyclohexane, 1*R*,2*R*-DACH) as carrier ligand. Oxaliplatin could form DNA adducts with different conformation than those formed by cisplatin and carboplatin, resulting in a different spectrum of activity, and, in addition, could induce cell death by facilitating a T cell-dependent immune response and perturbing ribosome biogenesis [3,4]. A common feature of these drugs is the appearance of resistance and induction of severe side effects that limit their use [5]. In the search for alternatives capable of bypassing the resistance; the Pt(II) complex [PtCl_2_(*cis*-1,4-DACH)], also named kiteplatin, carrying a structural isomer of the 1*R*,2*R*-diaminocyclohexane ligand present in oxaliplatin, has emerged as a valid alternative encompassing a very promising anticancer activity [6]. Kiteplatin is active against cisplatin-resistant ovarian C13* and oxaliplatin-resistant colon LoVo-OXP cell lines, suggesting that its spectrum of activity could be different from those of cisplatin and oxaliplatin. Furthermore, it was found that the 1,2-GG intrastrand crosslinks formed by kiteplatin are removed by DNA repair systems with lower efficiency than those formed by cisplatin and are more effective in inhibiting DNA polymerases such as the model prokaryotic DNA polymerase I (KF^−^) of the A-family and the eukaryotic translesion DNA polymerase η (Pol η) of the Y-family human DNA polymerases [7,8,9].

The above results prompted us to further investigate how limited structural variation on the diamino carrier ligand could impact the spectrum of activity of the platinum drugs. The chosen ligand was *trans*-1,2-diamino-4-cyclohexene (DACHEX), in which the introduction of a double bond in the cyclohexane ring greatly reduces its flexibility while increasing its planar extension. Four Pt(II) complexes: [PtCl_2_(DACHEX)], [PtI_2_(DACHEX)], [Pt(CBDCA)(DACHEX)], and [Pt(OXA)(DACHEX)] (CBDCA = 1,1′-cyclobutanedicarboxylate, OXA = oxalate), possessing the racemic diamine carrier ligand, were synthesized and tested against a panel of human tumor cell lines, including cervical (A431), ovarian (2008), and colon carcinomas (HCT-15 and LoVo), together with two oxaliplatin-resistant clones (LoVo OXP and LoVo MDR). The complexes proved to have, in general, equal if not better cytotoxic activity than cisplatin and oxaliplatin and were able to overcome the oxaliplatin-resistance. Moreover, the oxalate-derivative induced lipid droplets formation in LoVo OXP cells thus suggesting the involvement of metabolic stress in its mechanism of action [10].

In the present paper, we have expanded the investigation to Pt(IV) derivatives of [Pt(OXA)(DACHEX)]. Many studies have shown that Pt(IV) complexes have better pharmacological efficacy combined with a greater cellular uptake due to their increased lipophilicity [11,12,13]. In addition, the choice of the two additional axial ligands makes it possible to modulate the electrochemical properties of the complexes (particularly the reduction potential from Pt(IV) to Pt(II)) and, at the same time, to exploit the “drug targeting and delivery” concept through a careful selection of the ligands’ functional groups [14,15]. Furthermore, Pt(IV) prodrugs can be amenable to oral administration thanks to their greater stability and solubility in water [16].

In this paper, we describe the synthesis, structural characterization, redox behavior, and cytotoxic activity towards a panel of human tumor cell lines of six Pt(IV) complexes with the racemic DACHEX ligand: *cis,trans,cis*-[Pt(OXA)(OH)_2_(DACHEX)] (**1**), *cis,trans,cis*-[Pt(OXA)(AcO)_2_(DACHEX)] (AcO = acetate) (**2**), *cis,trans,cis*-[Pt(OXA)(BzO)_2_(DACHEX)] (BzO = benzoate) (**3**), *cis,trans,cis*-[Pt(OXA)Cl_2_(DACHEX)] (**4**), *cis,trans,cis*-[Pt(OXA)(AcO)Cl(DACHEX)] (**5**), and *cis,trans,cis*-[Pt(OXA)(OH)Cl(DACHEX)] (**6**).

## 2. Results

### 2.1. Synthesis and Characterization of Complexes 1–6

**Scheme 1:**
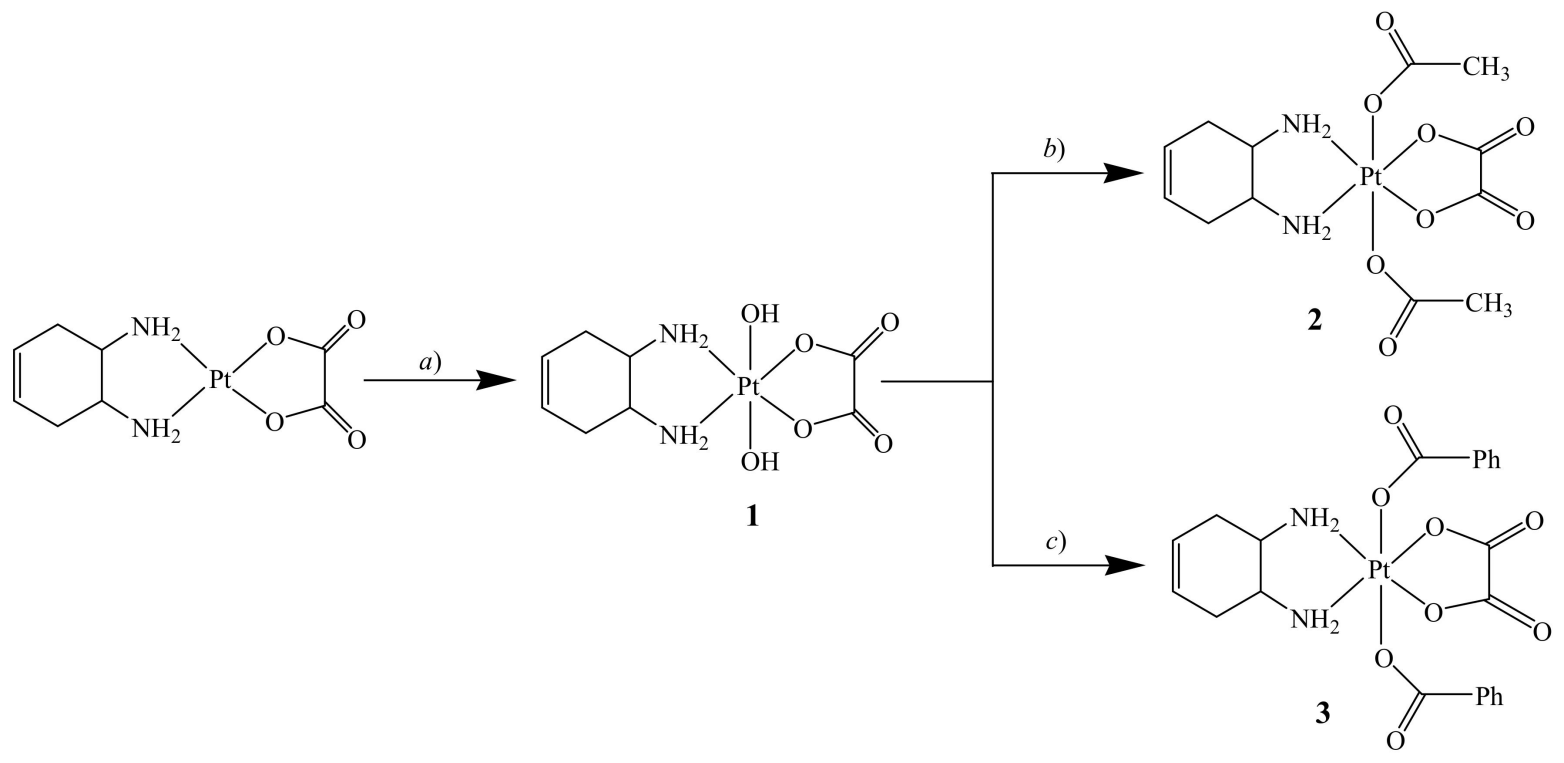
Synthesis of cis,trans,cis-[Pt(OXA)(OH)_2_(DACHEX)] (**1**), *cis*,*trans*,*cis*-[Pt(OXA)(AcO)_2_(DACHEX)] (AcO = acetate) (**2**), and *cis*,*trans*,*cis*-[Pt(OXA)(BzO)_2_(DACHEX)] (BzO = benzoate) (**3**). a) Excess H_2_O_2_, 70 °C, 2 h in the dark; b) Ac_2_O, 80 °C, 16 h in the dark; c) Acetone, Pyridine, PhC(O)Cl, 4 h reflux in the dark.

Complexes **1**–**3** were prepared by standard oxidation reaction of Pt(II) complexes followed by conjugation reactions [11,12,13,16,17,18]. The complex *cis,trans,cis*-[Pt(OXA)(OH)_2_(DACHEX)] (**1**) was synthesized by H_2_O_2_ oxidation of the Pt(II) precursor [Pt(OXA)(DACHEX)] (Scheme 1).

Complex **1** was isolated and characterized by NMR spectroscopy and electrospray ionisation mass spectrometry (ESI-MS). The ESI-MS spectrogram of compound **1** shows a molecular peak at *m*/*z* = 452.0372, assigned to the species [**1**+Na]^+^. The spectrum was consistent with the isotopic pattern predicted for [C_8_H_14_N_2_O_6_PtNa]^+^ (data not shown).

**Figure 1:**
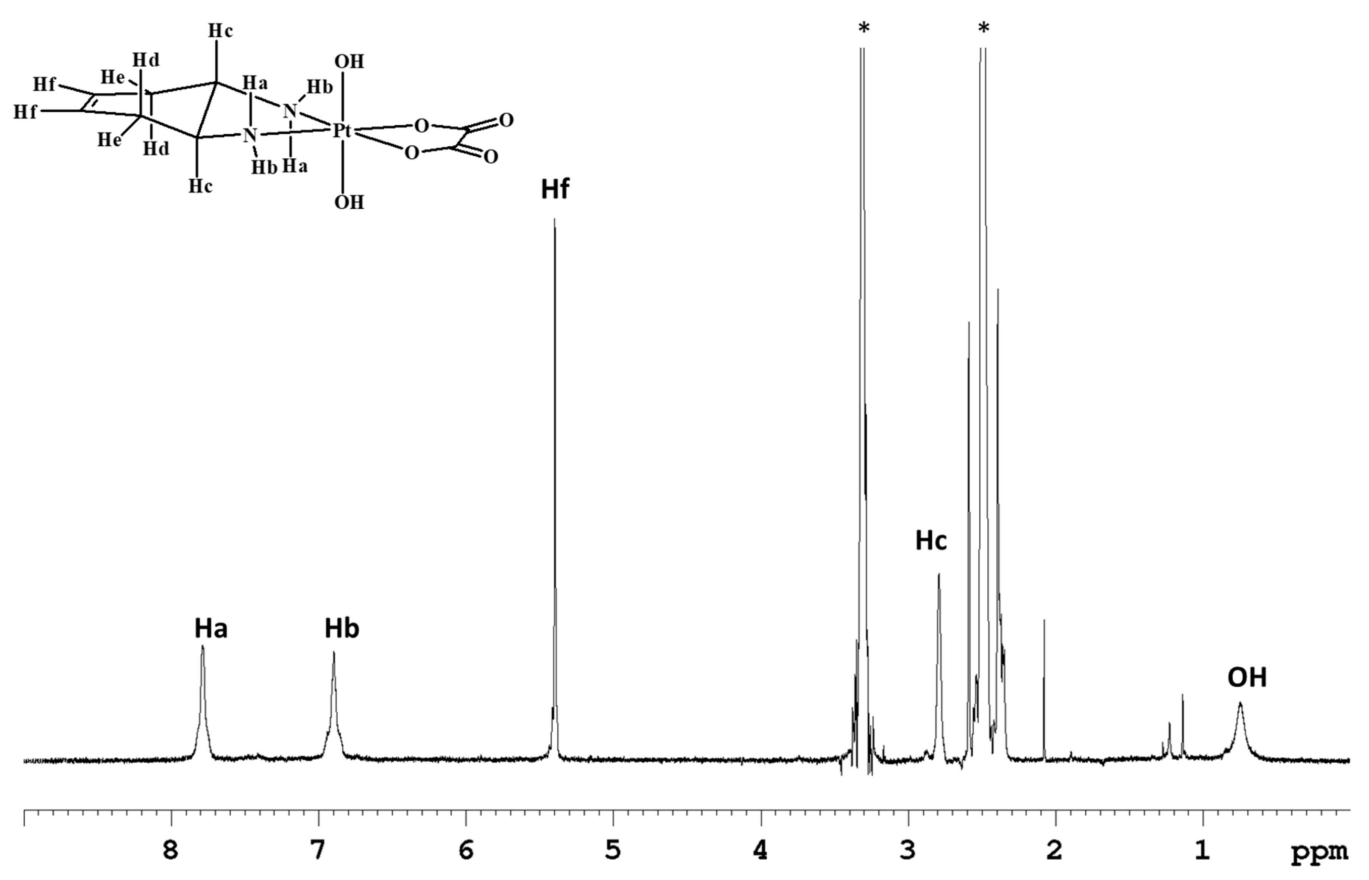
^1^H-NMR (700 MHz, ^1^H) of **1** in DMSO-d_6._ * indicates solvent residual peaks.

The ^1^H-NMR spectrum in deuterated dimethylsulfoxide (DMSO-d_6_) of complex **1** (Figure 1) shows two singlets, at 7.79 and 6.91 ppm, assigned to the amino protons in pseudo-axial (H_a_) and pseudo-equatorial (H_b_) positions, respectively (Table 1). The amino protons resonate at lower fields than the corresponding protons in the Pt(II) precursor [Pt(OXA)(DACHEX)] (6.27 and 5.49 ppm); the deshielding is a consequence of the change in the oxidation state of platinum. The 5.4 ppm singlet (integrating for two protons) was assigned to the vinylic protons (H_f_), while the signal at 2.80 ppm, integrating for two protons, was assigned to the methynic protons (H_c_). From the NMR spectrum in DMSO-d_6_, it was not possible to assign the methylene protons H_e_ and H_d_ due to overlap with the solvent signal. Finally, the broad signal at 0.74 ppm was assigned to the hydroxyl groups present in the axial positions.

For the characterization of the methylene protons, the ^1^H-NMR spectrum was recorded in D_2_O (Figure S1). The NMR characterization of complex **1** was completed by assignment of the ^13^C chemical shifts (Supplementary Figure S2 and Table 2).

The complex *cis,trans,cis*-[Pt(OXA)(AcO)_2_(DACHEX)] (**2**) was synthesized by *O*-acylation of complex **1** with acetic anhydride (Scheme 1). Carboxylate ligands in the axial positions of a Pt(IV) complex generally confer a reduction cathodic potential optimal for pharmacological application. In fact, the Pt(IV) complex was stable in the blood stream, but capable of undergoing reduction in the tumor environment where higher concentrations of reducing agents, such as glutathione and ascorbic acid, are present.

**Figure 2:**
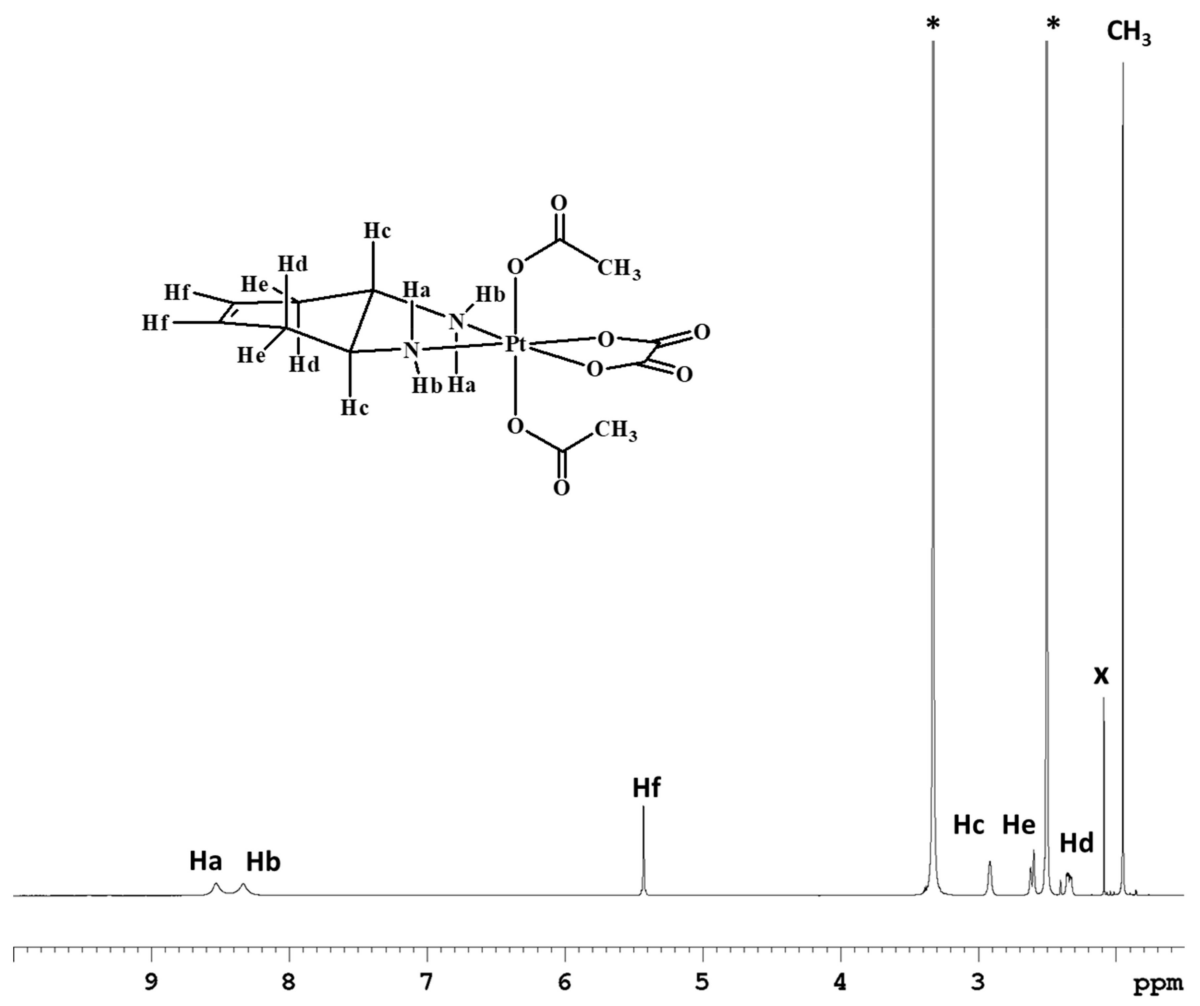
^1^H-NMR spectrum (700 MHz, ^1^H) of **2** in DMSO-d_6._ * indicates solvent residual peaks, x indicates an impurity (acetone) present in the solvent.

Complex **2** was characterized by ESI-MS and NMR. The ESI-MS spectrum shows the presence of a peak at *m*/*z* = 536.0591, corresponding to [**2**+Na]^+^, whose isotopic pattern is in excellent agreement with the theoretical calculation (data not shown). The ^1^H-NMR spectrum of compound **2** in DMSO-d_6_ was similar to that of compound **1**, with the exception of an additional peak attributable to the acetate ligands resonating at 1.95 ppm and integrating for six protons (Figure 2).

Complex **2** was also characterized by ^1^H-NMR in D_2_O; chemical shift values are reported in the Experimental Section and in Table 1. 

The ^13^C-NMR spectrum of **2** was similar to that of complex **1**, except for the presence of two additional peaks falling at 178.3 and 22.9 ppm assigned to carboxylic and methylic carbon atoms of the acetate ligands, respectively (Figure S3).

**Figure 3:**
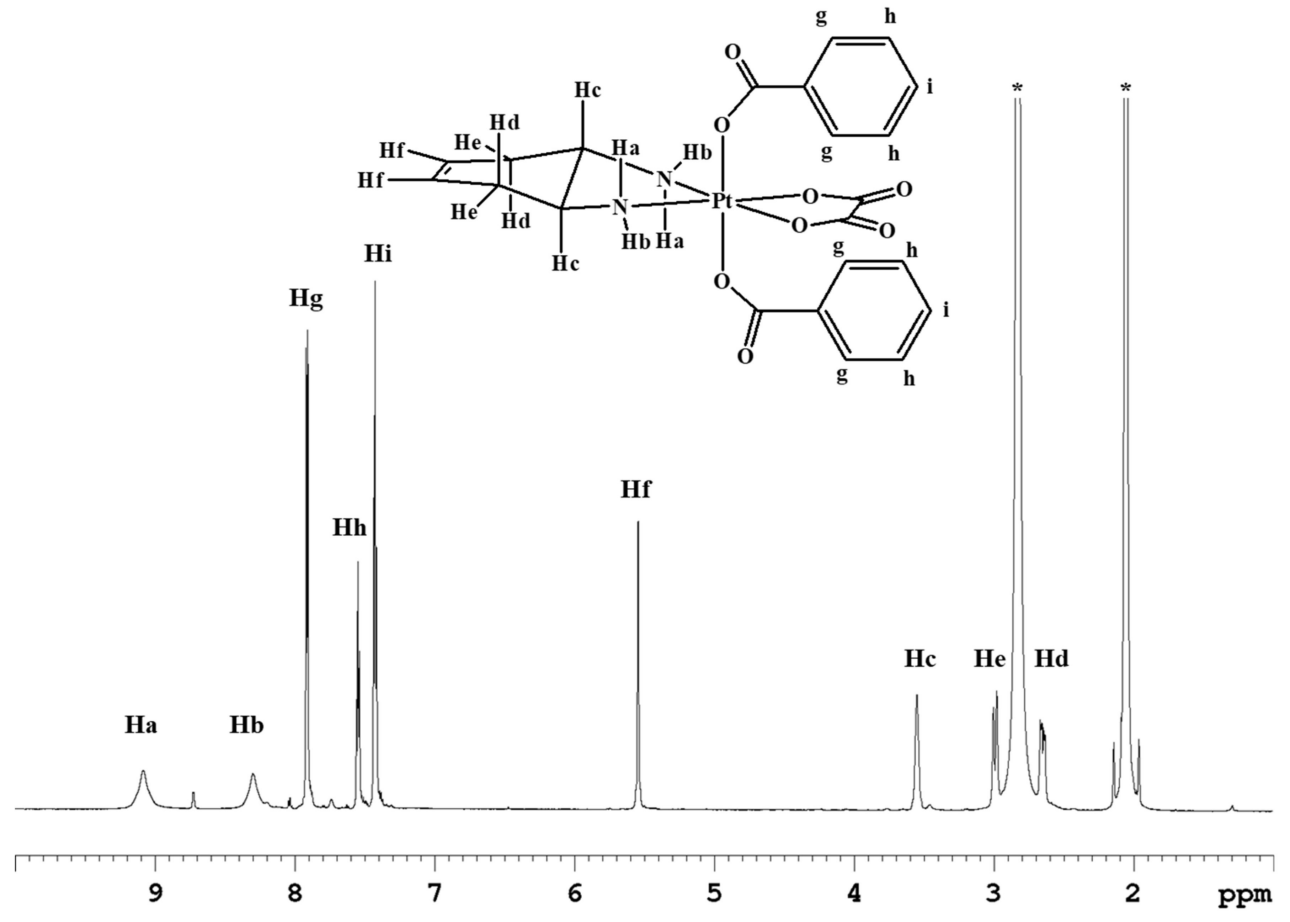
^1^H-NMR spectrum (700 MHz, ^1^H) of **3** in acetone-d_6._ * indicates residual solvent peaks.

The *cis,trans,cis*-[Pt(OXA)(BzO)_2_(DACHEX)] complex (**3**) contains benzoate axial ligands. Previous literature data have shown that the benzoate ligands in the axial positions of Pt(IV) derivatives of cisplatin and oxaliplatin confer pharmacological activity at nanomolar concentration, most likely because of the increased intracellular accumulation caused by passive diffusion through the cell membrane. [17,18] Complex **3** was synthesized by reacting complex **1** with benzoyl chloride in the presence of pyridine (Scheme 1). The ESI-MS spectrum of complex **3** showed the presence of a peak at *m/z* = 660.0889, corresponding to [**3**+Na]^+^, whose isotopic pattern is in good agreement with the calculated one (data not shown). The ^1^H-NMR spectrum of compound **3** in acetone-d_6_ (Figure 3) shows analogies with those of complexes **1** and **2**, but with the addition of aromatic signals due to the phenyl substituents. The signal resonating at 7.87 ppm (integrating for four protons) was assigned to the CH_g_ protons while the signals at 7.55 ppm (four protons) and 7.43 ppm (integrating for two protons) were assigned to CH_h_ and CH_i_ protons, respectively. The latter chemical shifts are quite similar to those observed for the analogous compounds *cis,trans,cis*-[PtCl_2_(BzO)_2_(*cis*-1,4-DACH)] (8.00, 7.57, and 7.47 ppm for protons CH_g_, CH_h_, and CH_i_, respectively)[18] and *cis,trans,cis*-[PtCl_2_(BzO)_2_(1*R*,2*R*-DACH)] (8.03, 7.52, and 7.38 ppm in CD_3_OD for protons CH_g_, CH_h_, and CH_i_, respectively) [17] having two chlorido ligands instead of an oxalate ligand in the equatorial positions.

The ^1^H NMR data are reported in Table 1, while Table 2 reports the ^13^C-NMR chemical shifts obtained from a spectrum recorded in acetone-*d_6_* (Figure S4).

**Scheme 2:**
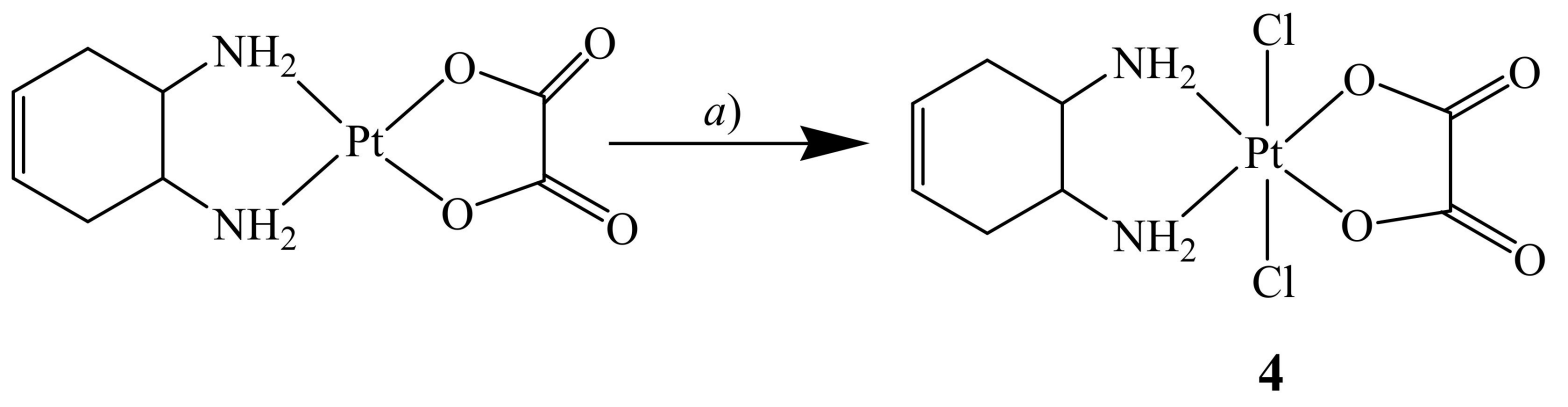
Synthesis of cis,trans,cis-[Pt(OXA)Cl_2_(DACHEX)] (**4**). a) CH_3_CN, PhICl_2_, 60 °C, 1 h.

The *cis,trans,cis*-[Pt(OXA)Cl_2_(DACHEX)] complex **4** was synthesized by reacting [Pt(OXA)(DACHEX)] with a solution of PhICl_2_ dissolved in acetonitrile (Scheme 2). The PhICl_2_ oxidant was synthesized by reaction of iodobenzene with chlorine and was preferred to molecular chlorine, as it is less aggressive.

Complex **4** was characterized by ESI-MS and NMR spectroscopy. The ESI-MS spectrum showed the presence of a peak at *m/z* = 488.9688, corresponding to [**4**+Na]^+^, whose isotopic pattern is in good agreement with the theoretical one (data not shown). The ^1^H-NMR spectrum of compound **4** dissolved in acetone-d_6_ is shown in Figure S5 and it is similar to that reported above for complex **1**. The chemical shift values are reported in the Experimental Section and in Table 1. 

Compound **4** proved to be quite unstable in DMSO-d_6_ solution and, after standing for 1 day at room temperature not shielded from the light, the ^1^H-NMR spectrum (Figure S6) showed the presence of new peaks assignable to the amino protons of the Pt(II) precursor complex [Pt(OXA)DACHEX)] (5.4–6.5 ppm). Most likely, compound **4** underwent a solvent-assisted reduction process favored by light, as previously observed for similar compounds such as *cis,trans,cis-*[PtCl_2_(OH)_2_(*cis*-1,4-DACH)] [19,20]. Furthermore, the presence of multiple sets of signals attributable to the amino protons of Pt(II)-coordinated DACHEX indicates that the reduction is accompanied by a solvation process. This reduction/solvation could also be observed in other solvents, such as D_2_O and CD_3_OD. The ^13^C NMR spectrum of **4** in acetone-d_6_ is reported in Figure S7 while the chemical shifts are reported in Table 2.

**Scheme 3:**
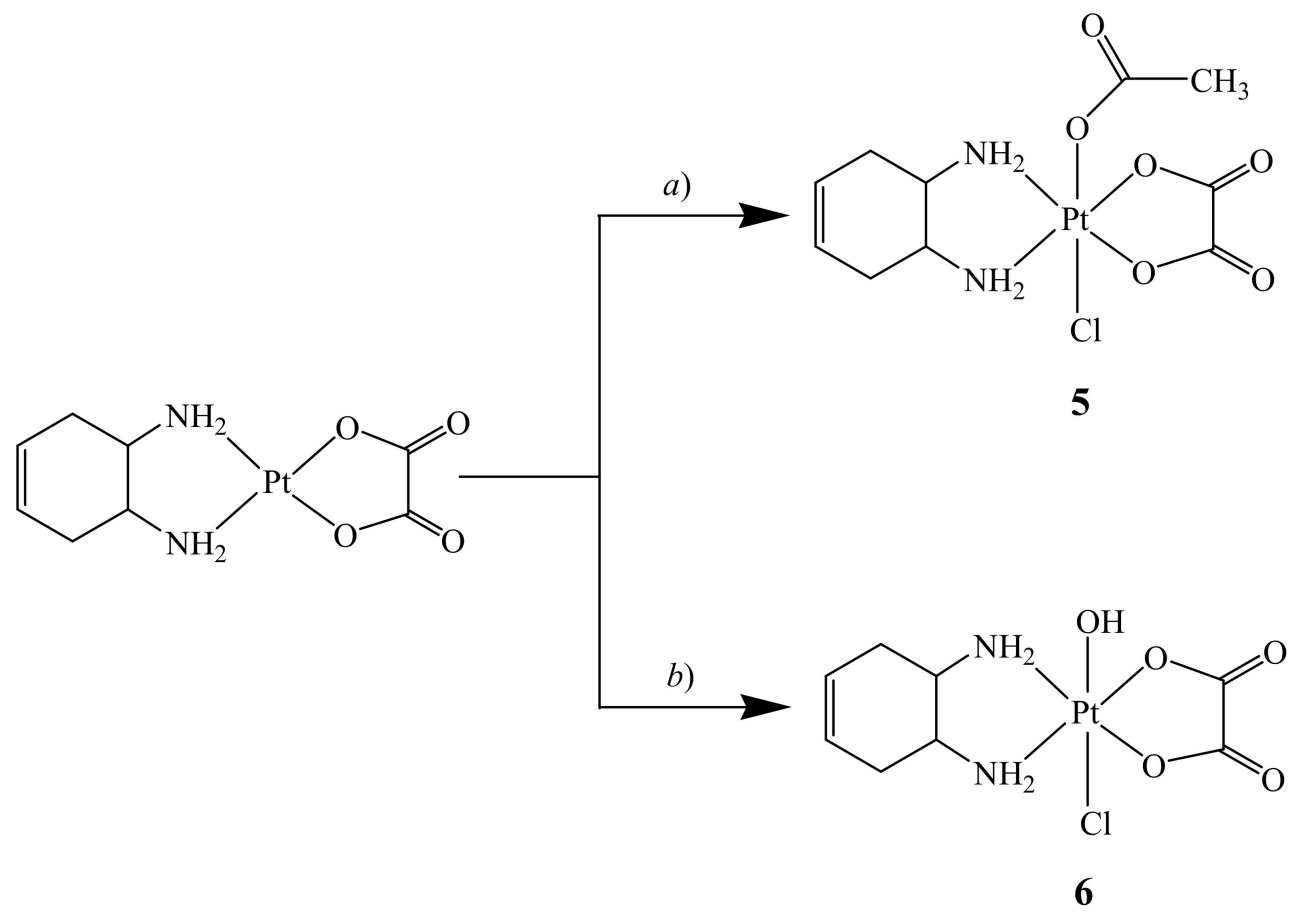
Synthesis of *cis,trans,cis*-[Pt(OXA)(AcO)Cl(DACHEX)] (**5**) and *cis,trans,cis*-[Pt(OXA)(OH)Cl(DACHEX)] (**6**). a) Acetic acid, *N*-chloro-succinimide, room temperature, dark, 4 h; b) water, *N*-chloro-succinimide, room temperature, dark, 3 h.

For synthesis of the asymmetric complexes *cis,trans,cis*-[Pt(OXA)(AcO)Cl(DACHEX)] (**5**) and *cis,trans,cis*-[Pt(OXA)(OH)Cl(DACHEX)] (**6**), a reliable synthetic approach, based on the employment of *N*-chloro-succinimide as oxidant, was used. Thus, addition of *N*-chloro-succinimide to the [Pt(OXA)(DACHEX)] complex dissolved in acetic acid leads to formation of compound **5** thanks to the large excess of acetic acid used as solvent which promotes the entry of the acetate into the second axial position (Scheme 3). In contrast, by addition of *N*-chloro-succinimide to [Pt(OXA)(DACHEX)] dissolved in water, compound **6** is formed (Scheme 3).

**Figure 4:**
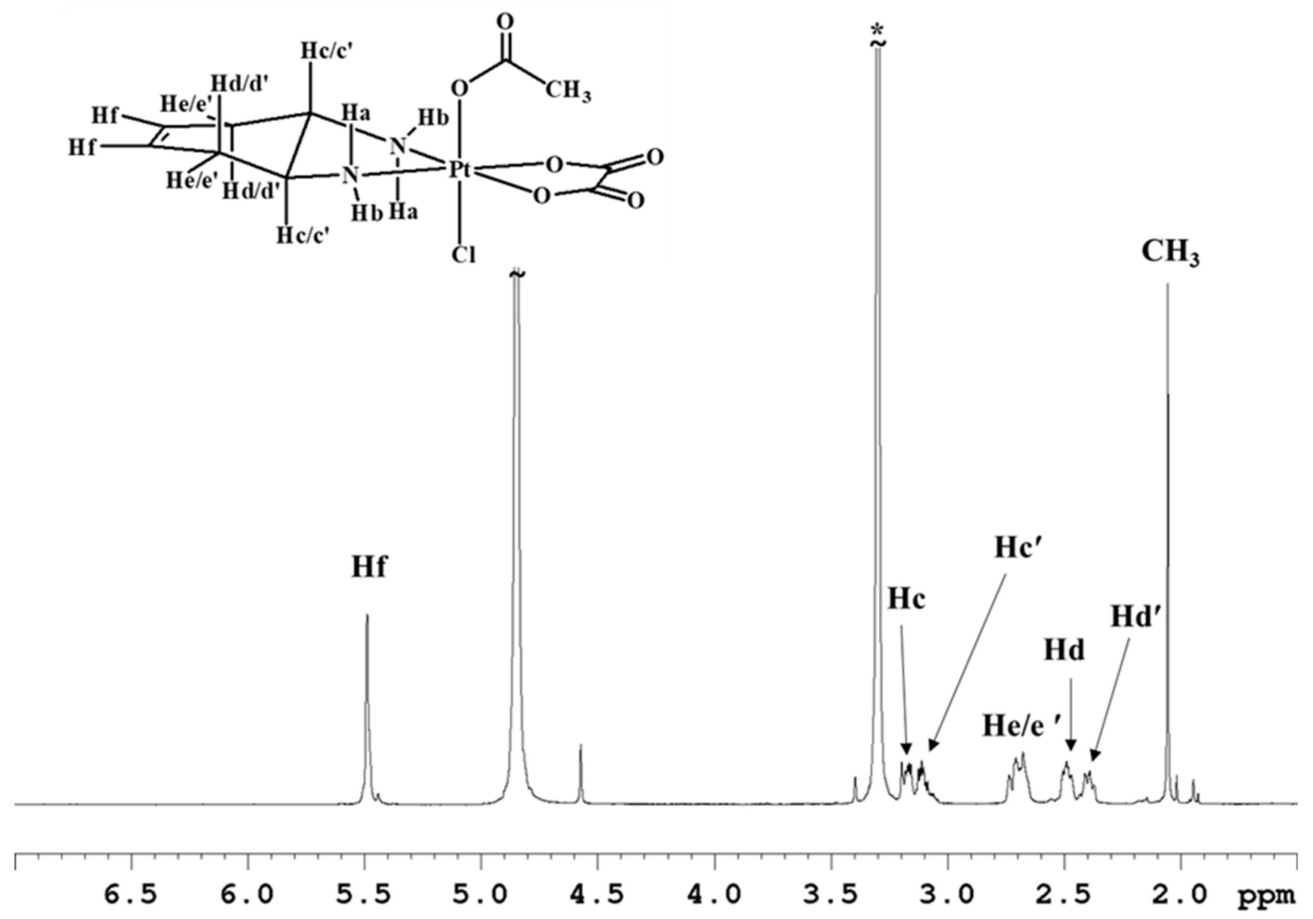
^1^H-NMR (700 MHz, ^1^H) spectrum of **5** in CD_3_OD. * indicates residual solvent peaks.

Complex **5** was characterized by ESI-MS and NMR spectroscopy. The spectrum in CD_3_OD (Figure 4 and Table 1) highlights the asymmetry of compound **5** as evidenced by splitting of the methynic and methylenic proton signals in two sets of signals. The two multiplets assigned to the methynic protons (H_c/c’_) fall at 3.17 and 3.11 ppm, while the four multiplets assigned to methylene protons fall two at 2.49 and 2.39 ppm (H_d/d’_ with quasi-axial orientation) and the other two overlap at 2.7–2.8 ppm (H_e/e’_ with quasi-equatorial orientation) (Figure 4). The amino protons were not detected in this spectrum due to the exchange with deuterium of the solvent.

The asymmetry of complex **5** also reflects on the ^13^C-NMR spectrum (Figure S8) showing doubling of some signals. Two peaks were detected for the vinyl carbons at 125.4 and 125.2 ppm; two signals at 60.3 and 60.2 ppm for the methynic carbons, and finally two peaks at 33.1 and 32.9 for the methylenic carbons. All the ^13^C chemical shift values are reported in Table 2. 

Similarly to compound **4**, compound **5**, also dissolved in DMSO-d_6_, shows a photo-assisted reduction accompanied by solvation of the resulting Pt(II) species. As for compound **4**, the reduction is probably fostered by the presence of axial chlorido ligand(s). 

Compound **6** was characterized by ESI-MS and NMR spectroscopy. The ESI-MS spectrum showed the presence of a peak at *m/z* = 471.0000 corresponding to [**6**+Na]^+^ with an isotopic pattern in good agreement with the theoretical one (data not shown). Similar to compound **5**, the ^1^H NMR spectrum of **6** in CD_3_OD was characterized by doubling of several signals due to the asymmetry of the compound with respect to the equatorial coordination plane (Figure S9 and Table 1). Two peaks related to the methynic protons fall at 3.13 and 3.07 ppm; two signals assignable to the quasi-axial methylenic protons fall at 2.48 and 2.42 ppm, while the quasi-equatorial methylenic protons give two signals overlapping at ca. 2.68 ppm.

**Figure 5:**
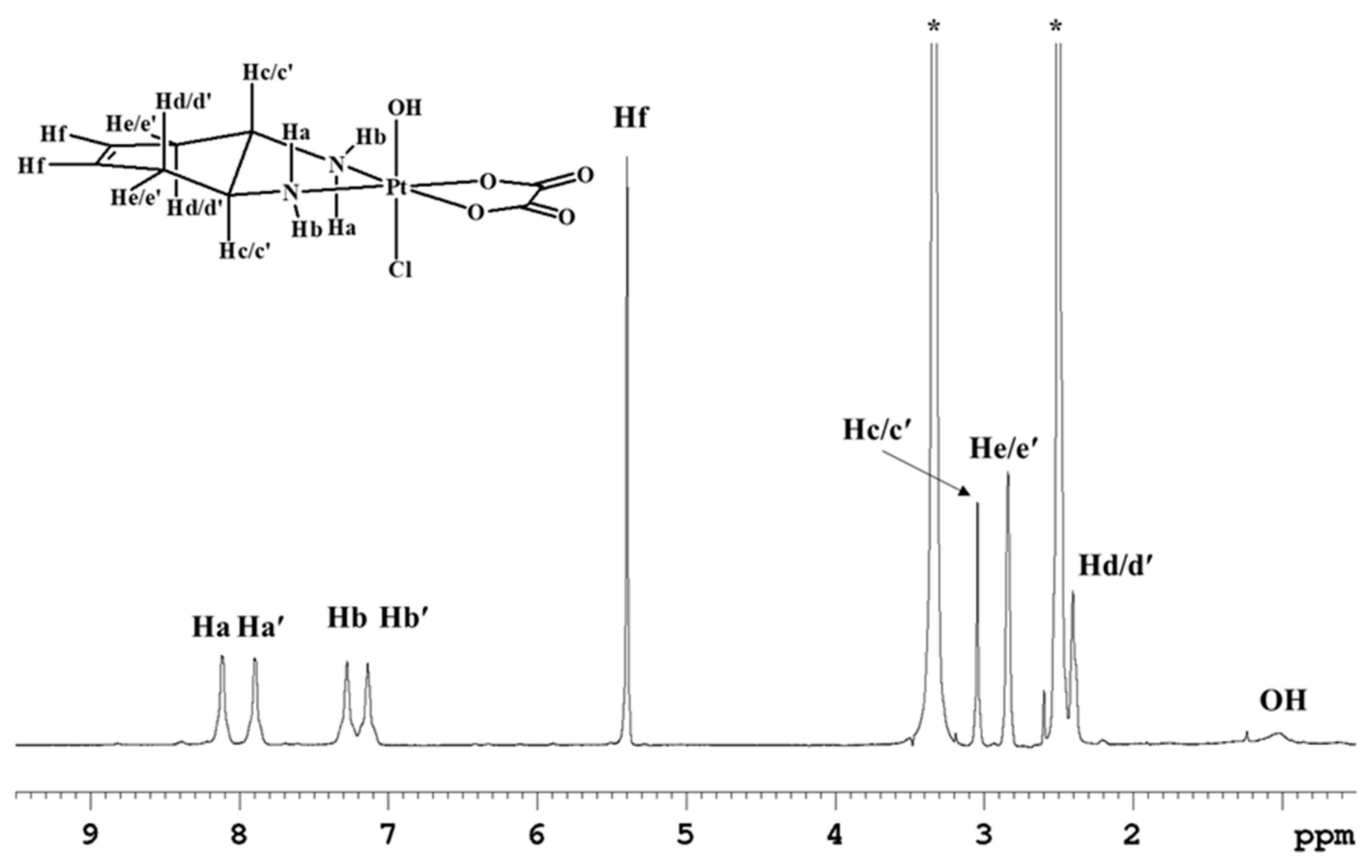
^1^H-NMR spectrum (700 MHz, ^1^H) of **6** in DMSO-d_6_. * indicates residual solvent peaks. The second signals relative to the methynic protons H_c/c’_ and to the methylenic protons H_d/d’_ are overlapping with solvent signals.

Complex **6** was also characterized by NMR in DMSO-d_6_ (Figure 5; Table 1) where the amino protons could be detected. Being an asymmetric complex with respect to the equatorial coordination plane, two signals falling at 8.11 and 7.89 ppm were observed for the quasi axial protons, and two signals falling at 7.27 and 7.15 ppm were observed for the quasi equatorial protons. Moreover, the broad signal that resonates at 1.03 ppm was assigned to the hydroxyl ligand in the axial position. 

Table 2 reports the ^13^C chemical shift values also for compound **6**. The ^13^C NMR signals are influenced by the asymmetry of the complex with respect to the equatorial plane with two peaks at 125.4 and 125.2 ppm for the vinylic carbons, two signals at 60.2 and 59.3 ppm for the methynic carbons, and two signals at 33.1 and 32.6 ppm for the methylenic carbons (Figure S10). Similar to **4** and **5**, complex **6** also underwent reduction followed by solvation in DMSO. 

### 2.2. Lipophilicity of the Complexes

Platinum(IV) pro-drugs have the advantage that their physicochemical properties can be tuned by choosing appropriate axial ligands. In particular, axial ligands will affect the lipophilicity and the reduction potential of the complex without affecting the DNA binding properties of the reduced bioactive platinum(II) species. Lipophilicity may be expressed in terms of the *n*-octanol/water partition coefficient (log*P*_o/w_), which can be determined by the classical shake-flask method or by HPLC techniques [21]. Usually, Pt(II) complexes are sufficiently hydrophilic to give negative log*P*_o/w_ values, while Pt(IV) complexes cover a wide range of lipophilicity showing either negative or positive log*P*_o/w_ values, the more positive values being associated to a higher cellular uptake of the complex. 

We determined the log*P*_o/w_ of the six Pt(IV) complexes and of the Pt(II) precursor [Pt(OXA)(DACHEX)] by the shake-flask method followed by inductively coupled plasma mass spectrometry (ICP-MS) determination of the Pt concentration in both the organic and the aqueous phases, for which the calculated log*P*_o/w_ values are reported in Table 3. Quite unexpectedly, the Pt(II) complex [Pt(OXA)(DACHEX)] resulted to be significantly more hydrophilic (log*P*_o/w_ = −2.43, Table 3) than oxaliplatin (log*P*_o/w_ = −1.76) [22]. We think that the π-electrons of the cyclohexene double bond increased the nucleophilicity and hence the hydrophilicity of [Pt(OXA)(DACHEX)] with respect to oxaliplatin. Given the high hydrophilicity of the DACHEX ligand, the log*P*_o/w_ values of all Pt(IV) complexes here investigated were negative (Table 3). As expected, the symmetric Pt(IV) complexes have log*P*_o/w_ values that decreased in the order **3** > **4** > **2** > **1** (−0.26, −1.65, −2.69, and −3.05, respectively; Table 3) in accordance with the decreasing order of lipophilicity of the coordinated axial ligands (benzoate > chloride > acetate > hydroxide, as simulated by the specific software Alogps 2.1; www.vcclab.org). The unsymmetrical complexes **5** and **6** had log*P*_o/w_ values of −2.03 and −2.39, which are also in line with the average lipophilicity of the corresponding axial ligands. It should be noted that only compounds **3**, **4**, and **5** were more lipophilic than the Pt(II) precursor complex [Pt(OXA)(DACHEX)], while compound **6** had nearly the same lipophilicity (log*P*_o/w_ of −2.39 to be compared with log*P*_o/w_ of −2.43 for [Pt(OXA)(DACHEX)]) and compounds **1** and **2** were less lipophilic than the Pt(II) precursor complex (log*P*_o/w_ of −3.05 and −2.69, respectively). 

### 2.3. Cyclic Voltammetry

**Figure 6:**
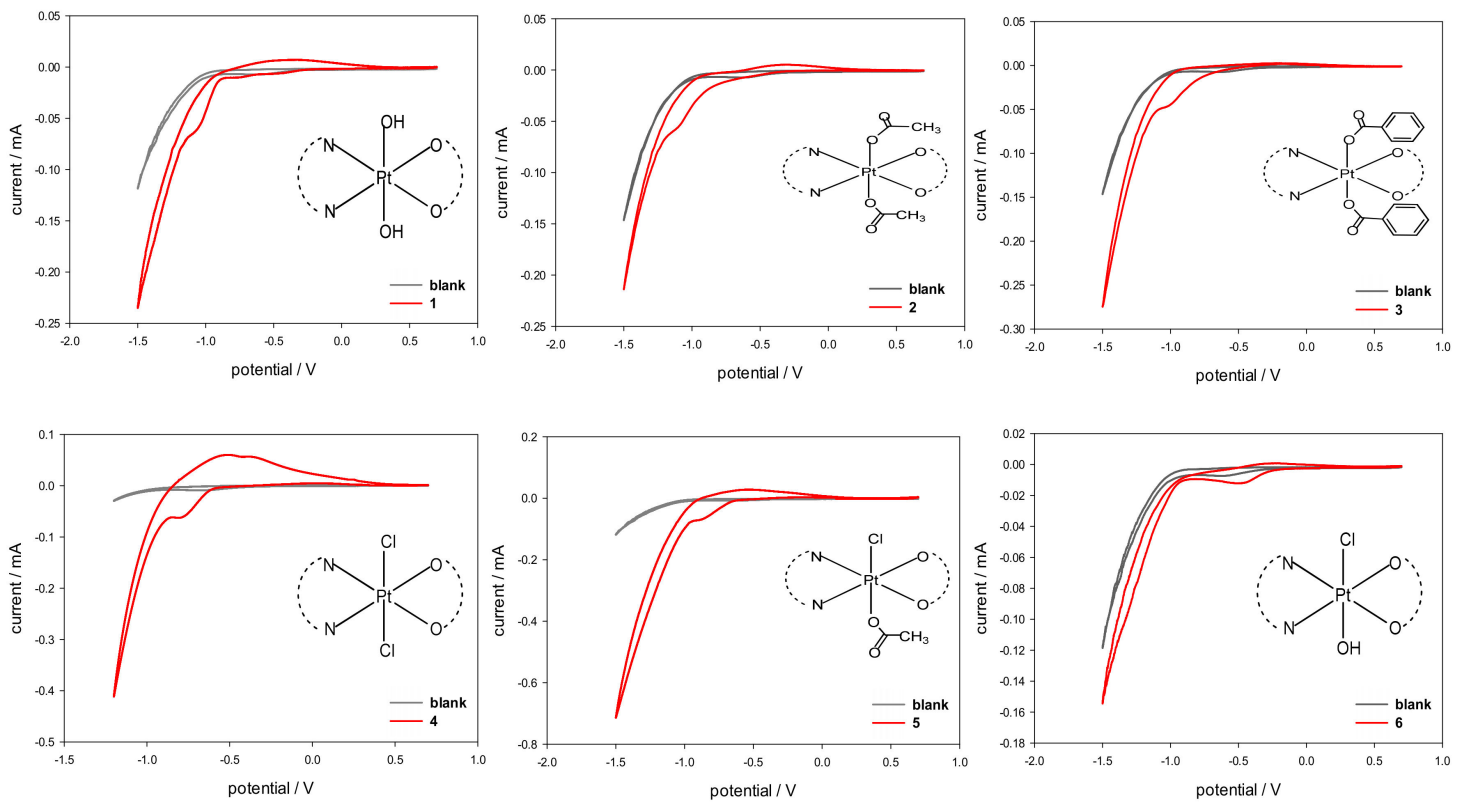
Cyclic voltammograms of Pt(IV) complexes **1**–**6** (red) recorded at a glassy carbon electrode in buffered aqueous solution (PBS, pH = 7.4, I = 0.1) containing 5 mM NaCl as supporting electrolyte and 5·10^−4^ M Pt(IV) complex. Scan rate 20 mVs^−1^. The CV plot of the blank solution is overlaid in grey.

The electrochemical characterization of compounds **1**–**6**, performed by using cyclic voltammetry (CV), was carried out with the aim of determining the cathodic reduction potentials in aqueous solution (E_p_^c^). The CV plots are reported in Figure 6 and the derived Ep^c^ values are listed in Table 3. Complexes **1**–**6** showed an electrochemical behavior typical of Pt(IV) complexes, with an irreversible reduction process (2e^−^) accompanied by a change in platinum coordination from octahedral (Pt(IV)) to square-planar (Pt(II)).

The obtained Ep^c^ values are in agreement with the values reported in the literature for complexes of Pt(IV) having a similar coordination environment [23,24,25,26,27,28].

In more detail, the reduction potentials appear to be influenced by the electron-withdrawing strength and the steric hindrance of the axial ligands. In the case of symmetrical complexes, the lowest reduction potential was found for compound **1** having two hydroxido ligands in axial positions (Ep^c^ = −1.05 V) while the highest reduction potential was observed for **4**, having two chlorido ligands in the axial positions (Ep^c^ = −0.72 V). An intermediate behavior, though shifted toward the lower limit, was observed for **2** and **3** obtained from **1** by substitution of the hydroxyl ligands with carboxylates (Ep^c^ of −1.03 and −0.99 V for **2** and **3**, respectively). As expected, the asymmetric complex **5**, having a chlorido and a benzoato ligand in axial positions, had an Ep^c^ value right in between those of **3** and **4**. To evaluate the effect of the electronegativity of the axial ligands on the cathodic reduction potentials, a linear regression was performed against the ‘total electronegativity’ χ_A(T)_ of the anionic ligands, as determined by Vaska et al. for a series of rhodium and iridium complexes (see Supplementary material Table S1) [29]. The χ_A(T)_ value includes the contributions of both the classical σ electronegativity and π acidity of the anionic ligand. For the asymmetric complexes, the average value for the two axial ligands was calculated. After excluding the hydroxido-chlorido complex **6**, the fitting showed a very good correlation (adjusted *R*^2^ = 0.94698) with the value of the average total electronegativity χ_A(T)_ (Supplementary material Figure S11). Indeed, unexpectedly, the asymmetric complex **6**, having one chlorido and one hydroxido substituent in the axial positions, had the highest reduction potential (Ep^c^ = −0.48 V), even greater than that of compound **4**. We are still investigating this unexpected result.

### 2.4. Characterization of the Surface Reduction

**Figure 7:**
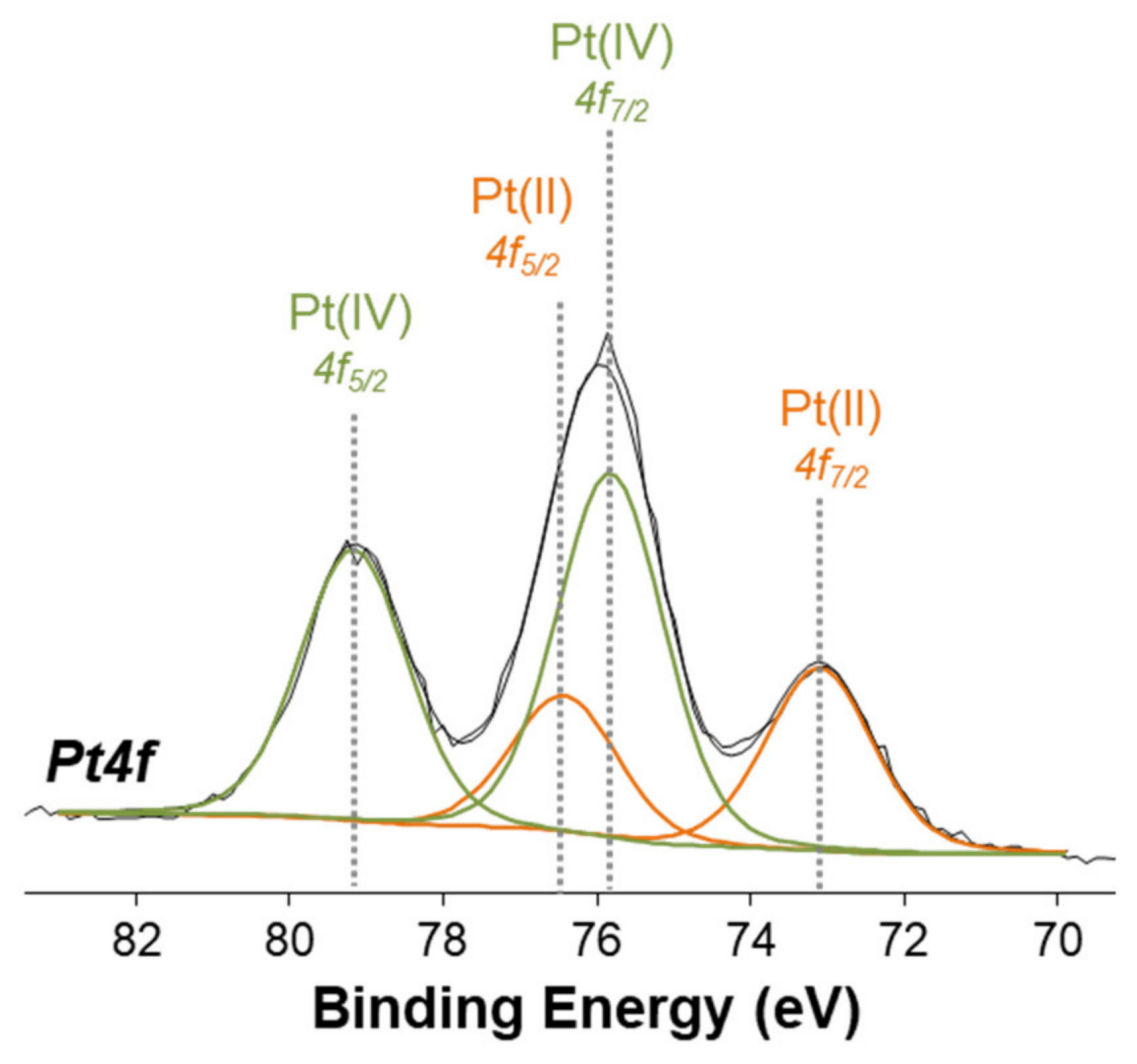
Pt4f XPS spectrum of complex **2**.

The Pt(IV) complexes were also characterized by X-ray photoelectron spectroscopy (XPS) in order to obtain information on the surface chemical composition and on the platinum speciation. Despite the surface sensitivity of the technique (topmost 5–8 nm), the analysis on homogeneous samples provides useful information on the chemical environment of all detected elements. Figure 7 shows the high resolution Pt4f XP spectrum of complex **2**, where the curve-fitting procedure has been applied to discriminate all the peak components. Two doublets were found: one is attributed to Pt(II) (BE Pt4f_7/2_ = 72.9 ± 0.2 eV and BE Pt4f_5/2_ = 76.2 ± 0.2 eV), while the second doublet is attributed to Pt(IV) (BE Pt4f_7/2_ = 75.6 ± 0.2 eV and BE Pt4f_5/2_ = 79.0 ± 0.2 eV).

The XPS spectrum clearly shows that some reduction of Pt(IV) to Pt(II) has taken place on the sample surface, while a sample of the same compound examined by NMR spectroscopy and ESI MS was shown to contain only the Pt(IV) species.

**Figure 8:**
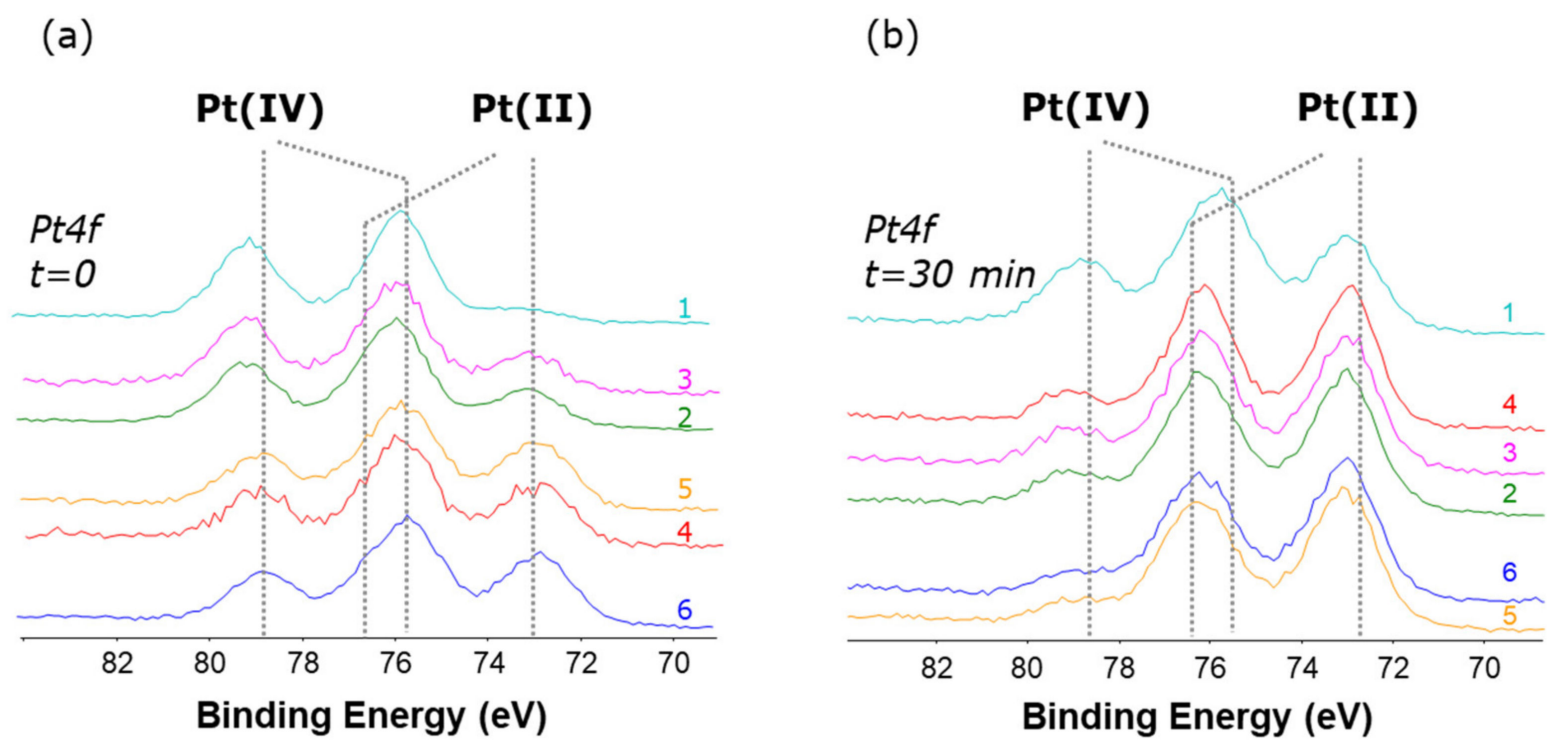
Pt4f XP spectra for complexes **1**–**6** (**a**) acquired at the onset of the experiments and (**b**) after 30 min irradiation. On each spectrum is shown the number that identifies the complex.

The same behavior was observed for all compounds **1**–**6** (overlays of the Pt4f XP regions for all compounds are shown in Figure 8a). The Pt(IV)/Pt(II) relative abundance varies from case to case; moreover, if the X-ray degradation is monitored after 30 min exposure to the X-ray beam, an increase of the Pt(II) relative abundance is observed (Figure 8b). These results clearly demonstrate the propensity of these Pt(IV) complexes to undergo reduction, and how the reduction occurring on the surface is dramatically enhanced by X-ray irradiation. 

Interestingly, both the initial Pt(IV)/Pt(II) relative abundance and the X-ray induced reduction were found to correlate with the reduction potentials obtained by cyclic voltammetry (Table 4). 

### 2.5. Cytotoxicity Assays

The in vitro cytotoxic activities of compounds **1**–**6** were assayed on a panel of human cancer cell lines containing examples of pancreatic (PSN-1), colon (HCT-15 and LoVo), cervical (A431), and ovarian (2008) cancers. Cisplatin and oxaliplatin were used as reference compounds and were tested under the same experimental conditions. The half maximal inhibitory concentration (IC_50_) values obtained by MTT (3-(4,5-dimethylthiazol-2-yl)-2,5-diphenyltetrazolium bromide) assay after 72 h of incubation, are reported in Table 5.

The new Pt(IV) compounds **1**–**6** were generally more effective than the reference compounds cisplatin (CDDP) and oxaliplatin (OXP). The mean IC_50_ values of all Pt(IV) complexes were remarkably lower (seven times) than that elicited by cisplatin and somewhat lower (2.5 times) than that of oxaliplatin. Compound **3**, with an average IC_50_ of 0.26 µM, was the most active: about five times more active than the other Pt(IV) species of the series, 33 times more active than cisplatin (average IC_50_ = 8.6 µM), and 12 times more active than oxaliplatin (average IC_50_ = 3.1 µM). These results were not completely unexpected, since it was known from the literature that complexes of Pt(IV) carrying benzoate ligands in the axial positions are very active, even at nanomolar concentrations, as it is in this case. [17,30] Thus, the pharmacological activity decreases in the order **3** >> **1** ≈ **2** ≈ **4** > **5** > **6**. Among all, the less active were the two unsymmetrical complexes **5** and **6** (Table 5). 

It is possible to correlate the cytotoxicity with the reduction potential and the log*P*_o/w_ values. Indeed, it can be noted how the most active complex **3** exhibited a reduction potential in the low range (Ep^c^ of −0.99, quite close to those of **1**, −1.05, and **2**, −1.03); however, compared to **1** and **2**, **3** had the greatest lipophilicity (log*P*_o/w_ of −0.26 to be compared with log*P*_o/w_ of −3.05 and −2.69 for **1** and **2**, respectively). Compound **4** exhibited a reduction potential in the high range (Ep^c^ of −0.72, intermediate between those of **5**, −0.82, and **6**, −0.48), but its lipophilicity (log*P*_o/w_ of −1.65) was second only to that of compound **3** and was greater than that of compounds **5** and **6** (log*P*_o/w_ of −2.03 and −2.39, respectively); as a consequence, **4** had greater cytotoxicity than **5** and **6**. The cytotoxicity of **4** was comparable to those of **1** and **2**, which had a lower reduction potential but also lower lipophilicity than **4**. 

All these results suggest that a low reduction potential and high lipophilicity are essential for good cytotoxicity. Indeed, as also seen for satraplatin, good activity is observed when the bioreduction of the pro-drug occurs inside the tumor cells and not in the circulatory stream. In addition, as observed in the case of **3**, a non-negligible contribution to cytotoxicity comes also from the lipophilicity that favors entering of the drug into the tumor cells by passive diffusion.

The antiproliferative activity was also investigated in LoVo-OXP cancer cells (Table 5), which were selected for their resistance to oxaliplatin. The main molecular mechanisms involved in oxaliplatin resistance appears to depend upon: (i) decreased cellular accumulation, which is thought to be related to a greater activity of the ATP7B exporter rather than to the activity of P-glycoprotein (P-gp) and multidrug resistance protein 1 (MRP1), and (ii) more efficient repair of oxaliplatin-induced DNA-damage by NER (Nucleotide Excision Repair) [17]. Interestingly, complex **3** was equally effective against sensitive (LoVo) and resistant (LoVo-OXP) colon cancer cells, thus attesting its remarkable ability to overcome the oxaliplatin resistance.

The remarkable cell-killing effect observed against human A431 squamous cervical carcinoma two-dimensional (2D) cultured cells prompted us to evaluate the in vitro antitumor activity of compounds **1**–**6** on three-dimensional (3D) cell cultures. As opposed to 2D monolayer cultures, cells growing in 3D culture systems form spheroids that comprise cells in various stages. The outer layers of the spheroid are highly exposed to the medium, and hence, are mainly comprised of viable, proliferating cells, whereas the core cells receive less oxygen, growth factors, and nutrients, and tend to be in a quiescent or hypoxic state [31]. Such cellular heterogeneity resembles that of in vivo tumors, making 3D cell cultures more predictive than conventional 2D monolayer cultures in screening antitumor drugs. Table 6 summarizes the IC_50_ values obtained after treatment of 3D cell spheroids of human A431 cervical cancer cells with the new Pt(IV) complexes as well as CDDP and OXP, used as reference compounds. All Pt(IV) compounds resulted to be much more effective against human A431 cancer cell 3D spheroids than CDDP and OXP and, consistently with 2D studies, complex **3** proved to be the most effective compound, showing an efficacy (in decreasing cancer spheroid viability) of about 32 and 20 times higher than those of CDDP and OXP, respectively.

**Figure 9:**
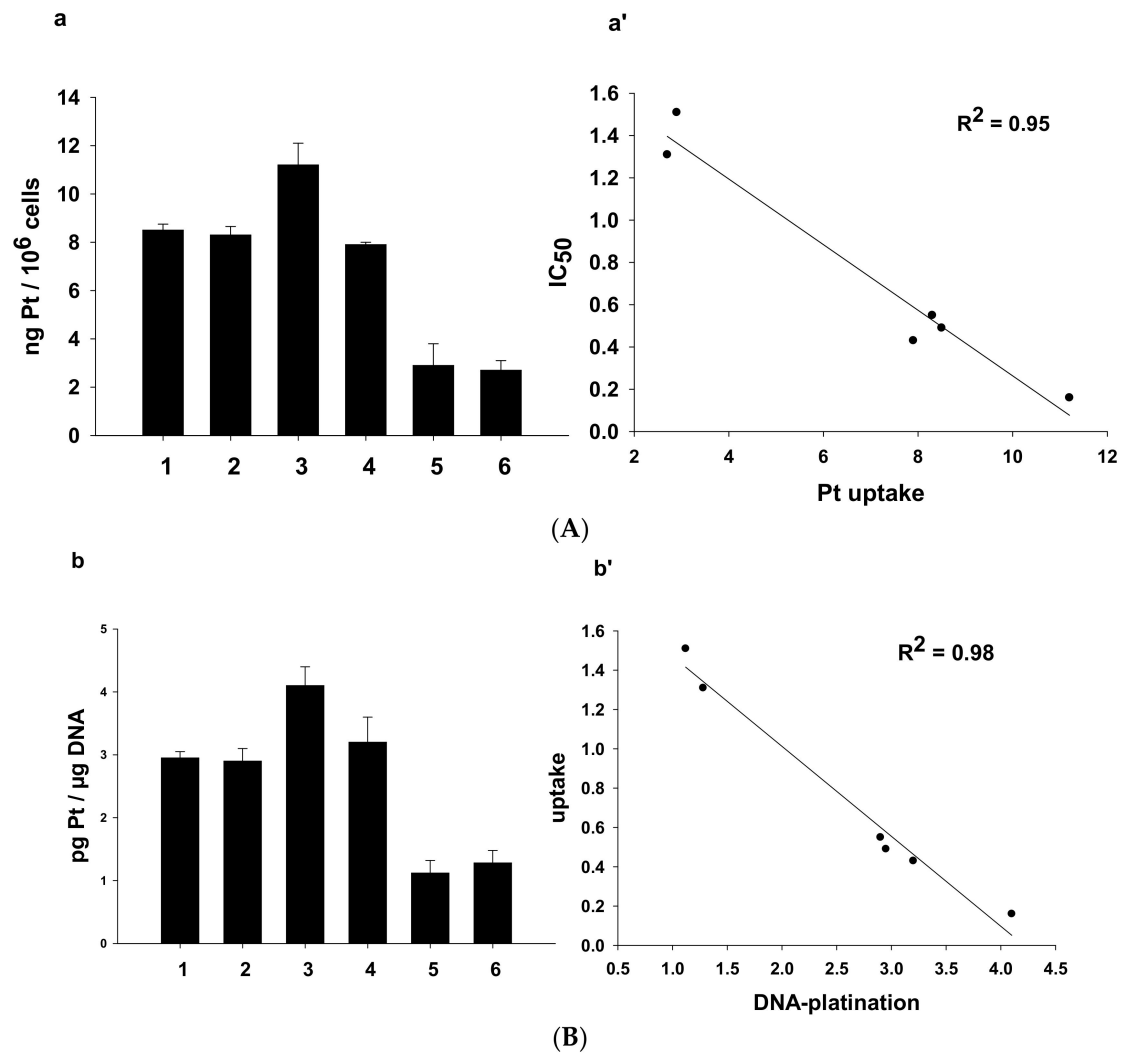
(**A**). Cellular uptake (a) and correlation between cytotoxicity and cellular platinum levels (a’) in treated A431 cancer cells. A431 cells were incubated for 24 h with 0.5 µM of **1**–**6**. The error bars indicate standard deviation (SD). (**B**). Nuclear DNA platination levels (b) and correlation between cellular uptake and DNA-platination levels (b’) in drug-treated A431 cells. A431 cells were treated for 24 h with 0.5 µM of **1**–**6**. DNA was extracted and quantified, and the amount of Pt bound to DNA was estimated using graphite furnace atomic absorption spectrometry (GF–AAS). Error bars indicate SD.

It is clearly recognized that the antitumor activity of platinum-based anticancer drugs depends on the ability of compounds to accumulate into cancer cells and to bind to nuclear DNA, thus inducing transcription inhibition and cell death [32]. Therefore, we investigated the cellular uptake as well as the DNA binding ability of **1**–**6** in human A431cervical cancer cells. Cells were treated for 24 h with 0.5 μM of **1**–**6** or CDDP and the total cellular and DNA-bound platinum contents were quantified using GF–AAS analysis. The results, expressed as ng of metal per 10^6^ cells, in the case of cellular uptake (A) and as pg of metal per μg of DNA, in the case of DNA-bound platinum (B) are summarized in Figure 9.

Both Pt(IV) asymmetric derivatives **5** and **6** were the worst in crossing the cell plasmalemma. 

Interestingly, the cellular internalization of **1**, **2,** and **4** was similar for the three complexes. Finally, the most cytotoxic bis-benzoate compound **3** was also the most effective in entering cancer cells.

By comparing uptake results with cytotoxicity data, a direct and linear correlation was found between the cytotoxic activity and the cellular uptake (plot a’ in Figure 9, *R*^2^ = 0.95). Similarly, a linear correlation was found between cellular uptake and DNA platination levels (plot b’ in Figure 9, *R*^2^ = 0.98). However, no correlation was found between log*P*_o/w_ and cellular uptake or between log*P*_o/w_ and reduction potential (Figure S12).

## 3. Materials and Methods 

### 3.1. Starting Materials and Instrumental Details

Commercial reagent grade chemicals and solvents (Sigma-Aldrich, Milan, Italy) were used as received without further purification. One-dimensional (1D) ^1^H-NMR, 1D ^13^C-NMR, and [^1^H-^13^C] HSQC 2D NMR spectra were recorded on Bruker Avance III 700 MHz and Bruker Avance DPX 300 MHz instruments (Bruker Italia S.r.L., Milano, Italy). ^1^H and ^13^C chemical shifts were referenced using the internal residual peak of the solvent (DMSO-d_6_: 2.50 ppm for ^1^H and 39.51 ppm for ^13^C; D_2_O: 4.80 ppm for ^1^H, Acetone-d_6_: 2.05 ppm for ^1^H and 29.92 for ^13^C, CD_3_OD: 3.31 ppm for ^1^H and 49.15 ppm for ^13^C). Electrospray ionisation mass spectrometry (ESI-MS) was performed with an electrospray interface and an ion trap mass spectrometer (1100 Series LC/MSD Trap system Agilent, Palo Alto, CA). 

Inductively Coupled Plasma Atomic Emission analyses were performed with an ICP-AES ThermoScientific iCap 6000 Series spectrometer (ICAP 6300 with dual view, empowered by iTeva software; Thermo Scientific, Waltham, MA, USA).

[Pt(OXA)(DACHEX)] and PhICl_2_ [10] were synthesized according to previously reported procedures.

### 3.2. Synthesis and Characterization of Platinum Complexes

#### 3.2.1. *cis,trans,cis*-[Pt(OXA)(OH)_2_(DACHEX)] (**1**)

[Pt(OXA)(DACHEX)] (114 mg, 0.288 mmol) was suspended in 74 mL of water. A solution of H_2_O_2_ in H_2_O (30% *w*/*w*, 1.36 mL) was added to the suspension and the reaction mixture was stirred in the dark at 70 °C for 2 h in an open flask. The resulting solution was cooled and dried under vacuum and the residue was treated with a mixture of acetone and diethyl ether (9:1, *v*/*v*, 10 mL) and kept under magnetic stirring for about 1 h. The suspended solid was isolated by filtration of the mother liquor, washed with ether, and dried under vacuum. In order to remove possible traces of H_2_O_2_, the solid product was dissolved in about 50 mL of water and the solution kept at 40 °C under magnetic stirring for 2 h, sonicating every 15 min. The solution was finally dried under reduced pressure, affording a white solid. Yield 61% (72 mg, 0.17 mmol). ESI-MS^+^: *calculated* for C_8_H_14_N_2_O_6_PtNa, [**1**+Na]^+^: 452.0397. *found*: *^m^/_z_* 452.0372. ^1^H-NMR (D_2_O): 5.53 (2H, CH_f_), 3.23 (2H, CH_c_), 2.81 (2H, CH_e_), 2.51 (2H, CH_d_) ppm. ^1^H-NMR (DMSO-d_6_): 7.79 (2H, NH_a_; *J*_H-Pt_ = 56 Hz) 6.91 (2H, NH_b_; *J*_H-Pt_ = 65 Hz) 5.40 (2H, CH_f_), 2.80 (2H, CH_c_), 2.49 (2H, CH_e_), 2.37 (2H, CH_d_) ppm. ^13^C-NMR (DMSO-d_6_): 164.7, 124.3, 57.1, 30.9 ppm. The numbering of protons is reported in Figure 1.

#### 3.2.2. *cis,trans,cis*-[Pt(OXA)(AcO)_2_(DACHEX)] (AcO = acetate = OOCCH_3_) (**2**)

*cis,trans,cis-*[Pt(OXA)(OH)_2_(DACHEX)] (40 mg, 0.0932 mmol) was suspended in 6.7 mL of acetic anhydride and the reaction mixture, placed in a flask equipped with a condenser, was kept under magnetic stirring at 80 °C for 16 h. The suspension was then dried under reduced pressure and the residue, treated with a cold mixture of water and acetone (0.50 mL; 1:1, *v*/*v*), was separated by filtration of the mother liquor, washed with ether and dried. Yield 54% (26 mg, 0.051 mmol).

ESI-MS^+^: *calculated* for C_12_H_18_N_2_O_8_PtNa, [**2**+Na]^+^: 536.0609. *Found*: *^m^/_z_* 536.0591. ^1^H-NMR (D_2_O): 5.52 (2H, CH_f_), 3.31 (2H, CH_c_), 2.81 (2H, CH_e_), 2.50 (2H, CH_d_), 2.08 (6H, CH_3_) ppm. ^1^H-NMR (DMSO-d_6_): 8.53 (2H, NH_a_) 8.33 (2H, NH_b_) 5.43 (2H, CH_f_), 2.92 (2H, CH_c_), 2.61 (2H, CH_e_), 2.34 (2H, CH_d_), 1.95 (6H, CH_3_). ^13^C-NMR (DMSO-d_6_): 178.3, 163.3, 124.3, 57.8, 31.5, 22.9. The numbering of protons is reported in Figure 2.

#### 3.2.3. *cis,trans,cis*-[Pt(OXA)(BzO)_2_(DACHEX)] (BzO = benzoate = OOC-C_6_H_5_) (**3**)

*cis,trans,cis*-[Pt(OXA)(OH)_2_(DACHEX)] (50 mg, 0.121 mmol) was suspended in 4 mL of acetone. Pyridine was added to the suspension (0.403 mL, 5 mmol), followed by a solution of benzoylchloride (0.37 mL) in 6 mL of acetone. The reaction was refluxed, in the dark and under magnetic stirring, for 4 h. The reaction mixture was brought to room temperature and the white solid was recovered by filtration of the mother liquor, washed, in the given order, with *n*-pentane, abundant ice-cold water, and diethyl ether, and finally dried under vacuum. Yield 35% (27 mg, 0.043 mmol).

ESI-MS^+^: *Calculated* for C_22_H_22_N_2_O_8_PtNa, [**3**+Na]^+^: 660.0922. *Found*: *^m^/_z_* 660.0889. ^1^H-NMR (acetone-d_6_): 9.08 (2H, NH_a_), 8.29 (2H, NH_b_), 7.92 (4H, CH_g_), 7.55 (4H, CH_h_), 7.43 (2H, CH_i_), 5.55 (2H, CH_f_), 3.55 (2H, CH_c_), 2.99 (2H, CH_e_), 2.65 (2H, CH_d_) ppm. ^1^H-NMR (DMSO-d_6_): 8.69 (2H, NH_a_) 8.32 (2H, NH_b_), 7.87 (4H, CH_g_), 7.56 (4H, CH_h_), 7.45 (2H, CH_i_), 5.47 (2H, CH_f_), 3.13 (2H, CH_c_), 2.68 (2H, CH_e_); CH_d_ overlaps with residual solvent peak and was not determined. ^13^C-NMR (acetone-d_6_): 176.0, 163.6, 133.4, 133.3, 130.6, 129.1, 125.3, 60.1, 33.4 ppm. The numbering of protons is reported in Figure 3.

#### 3.2.4. *cis,trans,cis*-[Pt(OXA)Cl_2_(DACHEX)] (**4**)

Complex **4** was prepared by dropwise addition of PhICl_2_ (23.1 mg, 0.084 mmol, previously dissolved in 2 mL of acetonitrile) to a suspension of [Pt(OXA)(DACHEX)] (30 mg, 0.076 mmol) in 3 mL of acetonitrile. The mixture, placed in a flask equipped with a condenser, was kept under magnetic stirring at 60 °C for 1 h. The pale yellow precipitate that formed was filtered, washed with ether (3 × 0.2 mL), and dried under vacuum. Yield: 56% (19 mg, 0.04 mmol).

ESI-MS^+^: *calculated* for C_8_H_12_N_2_O_4_Cl_2_PtNa, [**4**+Na]^+^: 488.9671. *found*: *^m^/_z_* 488.9688. ^1^H-NMR (acetone-d_6_): 7.75 (2H, NH_a_), 6.98 (2H, NH_b_), 5.59 (2H, CH_f_), 3.47 (2H, CH_c_), 2.97 (2H, CH_e_), 2.13 (2H, CH_d_) ppm. ^1^H-NMR (DMSO-d_6_): 8.40 (2H, NH_a_) 7.69 (2H, NH_b_) 5.39 (2H, CH_f_), 2.87 (2H, CH_c_), 2.55 (2H, CH_e_), CH_d_ overlaps with the residual solvent signal and was not determined. ^13^C-NMR (acetone-d_6_): 163.4, 125.0, 60.0, 32.8 ppm. The numbering of protons is reported in Figure S5.

#### 3.2.5. *cis,trans,cis*-[Pt(OXA)(AcO)Cl(DACHEX)] (**5**)

Complex **5** was prepared by a modification of oxidative chlorination reactions reported in the literature. [33,34] *N*-chloro-succinimide (18 mg, 0.133 mmol) was dissolved in 2 mL of acetic acid, then added to a solution containing [Pt(OXA)(DACHEX)] (50 mg, 0.126 mmol) previously dissolved in 2 mL of the same solvent. The reaction mixture was kept at room temperature, in the dark, for 4 h. The obtained solution was taken to dryness under reduced pressure, and the residue was dissolved in methanol and purified by precipitation with diethyl ether. The obtained precipitate was isolated by filtration of the mother liquor, washed with ether, and dried under vacuum. Yield 41% (26 mg, 0.05 mmol).

ESI-MS^+^: *Calculated* for C_10_H_15_N_2_O_6_ClPtNa, [**5**+Na]^+^: 513.0116. *Found*: *^m^/_z_* 513.0143. ^1^H-NMR (CD_3_OD): 5.49 (2H, CH_f_), 3.17 (1H, CH_c_), 3.11 (1H, CH_c’_), 2.71 (2H, CH_e_), 2.49 (1H, CH_d_), 2.39 (1H, CH_d’_), 2.06 (3H, CH_3_) ppm. ^1^H-NMR (DMSO-d_6_): 8.52 (2H, NH_a_) 7.76 (2H, NH_b_) 5.41 (2H, CH_f_), 2.89 (2H, CH_c_), 2.59 (2H, CH_e_), 2.13 (2H, CH_d_), 1.97 (3H, CH_3_). ^13^C-NMR (CD_3_OD): 182.1 (acetate carboxylic carbon), 166.7, 125.4, 125.2, 60.3, 60.2, 33.1, 32.9, 23.9 (CH_3_) ppm. The numbering of protons is reported in Figure 4.

#### 3.2.6. *cis,trans,cis*-[Pt(OXA)(OH)Cl(DACHEX)] (**6**)

Complex **6** was prepared with a synthetic procedure similar to that reported for complex **5**. Briefly, a suspension of [Pt(OXA)(DACHEX)] (50 mg, 0.126 mmol) in 16 mL of water was treated with *N*-chloro-succinimide (18.6 mg, 0.139 mmol). The mixture was left under magnetic stirring for 3 h at room temperature in the dark and then filtered. The filtrate was concentrated to a minimum volume until incipient formation of a precipitate. The suspension was left standing overnight at 4 °C and the pale yellow precipitate was isolated by filtration of the mother liquor, washed with ether, and dried under vacuum. Yield 55% (31 mg, 0.06 mmol).

ESI-MS^+^: *Calculated* for C_18_H_13_N_2_O_5_ClPtNa, [**6**+Na]^+^: 471,0010. *Found*: *m*/*z* 471.0000. ^1^H-NMR (DMSO-d_6_): 8.11 (1H, NH_a_), 7.89 (1H, NH_b_), 7.27 (1H, NH_a’_), 7.14 (1H, NH_b’_), 5.40 (2H, CH_f_), 3.04 (2H, CH_c_), 2.84 (2H, CH_e_), 2.40 (2H, CH_d_), ppm. ^1^H-NMR (CD_3_OD): 5.48 (2H, CH_f_), 3.13 (1H, CH_c_), 3.07 (1H, CH_c’_), 2.68 (2H, CH_e_), 2.48 (1H, CH_d_) and 2.42 (1H, CH_d’_) ppm. ^13^C-NMR (CD_3_OD): 167.1, 125.4, 125.2, 60.2, 59.3, 33.1 (^3^*J*_C-Pt_ = 37.6 Hz), 32.6 (^3^*J*_C-Pt_ = 36.1 Hz) ppm. The numbering of protons is reported in Figure 5.

### 3.3. LogP_o/w_ Determination

The hydrophobicity (log*P*) of the series of compounds was determined using the shake-flask method. Aqueous (0.15 M KCl in MilliQ water, 100 mL) and organic (*n*-octanol, 100 mL) phases were mixed together for 72 h to allow saturation of both phases. The Pt compounds (*ca*. 1 mg) were dissolved in the aqueous phase (1.0 mL) at a final concentration of ca. 2 mM and an equal volume of saturated *n*-octanol was added. The two phases were mixed for 30 min using a magnetic stirrer set at 600 rpm. Samples were centrifuged (1500 g, 10 min) to separate the two phases and the platinum contents of the organic and aqueous phases were determined by ICP-MS. The value of log*P*_o/w_ was calculated as the logarithm of the ratio between the Pt concentration in the organic phase and the aqueous phase.

### 3.4. Electrochemical Measurements

Cyclic voltammetry (CV) measurements were carried out with a potentiostat CHI 1230B (CH Instruments, Inc., Austin, TX, USA) in a standard three-electrode electrochemical cell. The working electrode was a glassy carbon (GC) electrode, the reference electrode was a KCl-saturated Ag/AgCl and the counter was a Pt sheet. The GC electrode was carefully polished with alumina powder, then rinsed with distilled water and dried, to obtain a reproducible surface for all the experiments. All the measurements, conducted in triplicate, were carried out in buffered aqueous solutions containing the Pt(IV) compound at a concentration of 5·10^−4^ M in 10 mL phosphate buffer (PB, pH = 7.4, I = 0.1) and 5 mM NaCl as supporting electrolyte. A blank solution (without Pt(IV) complex) was also tested. All the peak potentials were measured with a scan rate of 0.020 V s^−1^; the initial potential was chosen equal to the open-circuit potential and the scan direction was negative. All the CV plots were acquired after an equilibration time of 2 min in order to make the GC surface reproducible. 

### 3.5. Surface Chemical Characterization

X-ray Photoelectron Spectroscopy (XPS) analyses were run on a PHI 5000 Versa Probe II Scanning XPS Microprobe spectrometer (ULVAC PHI Inc., Kanagawa, Japan). Measurements were carried out using a monochromatic Al Kα source (X-ray spot 200 μm) at a power of 50.3 W. Wide scan and detailed spectra (Pt4f, C1s, Cl2p, N1s, O1s) were acquired in Constant Analyzer Energy (CAE) mode with a pass energy of 117.40 and 29.35 eV, respectively. An electron gun was used for charge compensation (1.0 V, 20.0 μA). Due to the X-ray sensitivity of Pt(IV) complexes, the Pt4f spectral region was acquired first. X-ray degradation kinetics experiments were performed analyzing the same sample area after 30 min. Data processing was performed by using the MultiPak software v. 9.8.0.19.

### 3.6. Cellular Cultures

Human colon (HCT-15 and LoVo) and pancreatic (PSN-1) carcinoma cell lines were obtained from the American Type Culture Collection (ATCC, RocKville, MD). The 2008 human ovarian cancer cells were kindly provided by Prof. G. Marverti (Dept. of Biomedical Science of Modena University, Italy). Human cervical carcinoma (A431) cells were kindly provided by Prof. F. Zunino (Division of Experimental Oncology B, Istituto Nazionale dei Tumori, Milan, Italy). The LoVo-OXP cells were derived, using a standard protocol, by growing LoVo cells in increasing concentrations of oxaliplatin and following nine months of selection of resistant clones, as previously described [35]. Cell lines were maintained in the logarithmic phase at 37 °C in a 5% carbon dioxide atmosphere using RPMI-1640 or F-12 culture medium (Euroclone) containing 10% fetal calf serum (Euroclone, Milan, Italy), antibiotics (50 units per mL penicillin and 50 μg mL^−1^ streptomycin), and 2 mM L-glutamine.

#### 3.6.1. MTT Test

The growth inhibitory effect toward the tumor cell lines was evaluated by the MTT test. Briefly, 3-8·10^3^ cells/well, depending upon the growth characteristics of the cell lines, were seeded in 96-well microplates in growth medium (100 µL) and then incubated at 37 °C in a 5% carbon dioxide atmosphere. After 24 h, the medium was removed and replaced with a fresh one containing the compound to be tested at the appropriate concentration. Triplicate cultures were established for each treatment. After 72 h, each well was treated with 10 µL of a 5 mg·mL^−1^ MTT (3-(4,5-dimethylthiazol-2-yl)-2,5-diphenyltetrazolium bromide) saline solution and, after 5 h of incubation, 100 µL of a sodium dodecylsulfate (SDS) solution in HCl 0.01 M were added. Following an overnight incubation, the inhibition of cell growth induced by the tested complexes was evaluated by measuring the absorbance of each well at 570 nm using a Bio-Rad 680 microplate reader (Bio-Rad Laboratories S.r.l., Segrate (MI), Italy).

#### 3.6.2. Spheroid Cultures

Spheroid cultures were obtained by seeding 2.5 × 10^3^ A431 cancer cells/well in a round bottom non-treated tissue culture 96 well-plate (Greiner Bio-one, Kremsmünster, Austria) in phenol red free RPMI-1640 medium (Sigma Chemical Co.) containing 10% fetal calf serum (FCS) and supplemented with 20% methyl cellulose stock solution.

#### 3.6.3. Acid Phosphatase (APH) Assay

An APH modified assay was used for determining cell viability in 3D spheroids, as previously described. [36] IC_50_ values were calculated with a four parameter logistic (4-PL) model.

#### 3.6.4. Cellular Accumulation of Pt

A431 cells (2·10^6^) were seeded in 75 cm^2^ flasks in growth medium (20 mL). After 24 h, the medium was replaced and the cells incubated for 24 h in the presence of the tested complexes.

Cells monolayers were washed twice with cold phosphate-buffered saline (PBS), harvested, and counted. Samples were subjected to three freeze–thaw cycles at −80 °C, and then vigorously vortexed. The samples were treated with highly pure nitric acid (1 mL; Pt ≤ 0.01 mg kg^−1^, Trace-SELECT Ultra, Sigma Chemical Co.) and transferred into a microwave Teflon vessel. Subsequently, samples were submitted to standard mineralization procedures. Samples were analyzed for platinum by using a Varian AA Duo graphite furnace atomic absorption spectrometer (Varian, Palo Alto, CA; USA) at a wavelength of 324.7 nm. The calibration curve was obtained using known concentrations of standard solutions purchased from Sigma Chemical Co.

#### 3.6.5. DNA Platination

A431 cells (5∙10^6^) were seeded in 10 cm Petri dishes in 10 mL of culture medium. Subsequently, cells were treated with tested complexes for 24 h. DNA was extracted and purified by a commercial spin column quantification kit (Qiagen DNeasy Blood and Tissue Kit). Only highly purified samples (A260/A230 ≅ 1.8 and A280/A260 ≅ 2.0) were included for analysis to avoid any artefacts. The samples were completely dried and re-dissolved in 200 μL of Milli-Q water (18.2 MΏ) for at least 20 min at 65 °C in a shaking thermo-mixer, then mineralized and analyzed for total Pt content by GF-AAS as described above.

## 4. Conclusions

Based on the preliminary results obtained for the Pt(II) complex [Pt(OXA)(DACHEX)] (an oxaliplatin-analogue possessing a double bond in the 4-position of the 1,2-diaminocyclohexane ring), which proved to have a cytotoxic activity better than that of cisplatin and comparable to or better than that of oxaliplatin and to be equally active against LoVo and LoVo-OXP cells, in this paper we have extended the investigation to corresponding Pt(IV) derivatives having different ligands in the axial positions. The six new Pt(IV) complexes (**1**–**6**) were fully characterized by spectroscopic and spectrometric techniques and NMR studies revealed a solvent-assisted reduction taking place when at least one chlorido ligand is present in the axial position (compounds **4**, **5**, and **6**). By cyclic voltammetry, it was shown that all complexes have a cathode potential (E_p_^c^) compatible with an intracellular reduction by the common bio-reducing agents ascorbate, glutathione, and NADH (reduced nicotinamide adenine dinucleotide), with previously mentioned compounds **4**, **5**, and **6** having greater tendency to undergo reduction than compounds **1**–**3** having only hydroxido (**1**) or carboxylato (**2** and **3**) ligands in axial positions. XPS experiments also showed a surface reduction of Pt(IV) to Pt(II), which increases with X-ray irradiation and that neatly correlates with the E_p_^c^ values calculated from cyclic voltammetry.

Interestingly, compound **3** was found to be active at nanomolar concentrations. Its benzoate axial ligands confer to the complex high lipophilicity that plays a determining role in increasing the cytotoxicity as confirmed by cellular uptake and DNA-platination studies. Additionally, the potency of compound **3** was fully confirmed toward human A431 cancer cell spheroids whose cellular heterogeneity makes them more predictive for antitumor activity *in vivo*. Our aim is to confirm the results obtained with 3D cell spheroids in in vivo experiments.

## Figures and Tables

**Table 1:** ^1^H-NMR chemical shifts (ppm) for complexes **1**–**6** in different deuterated solvents.

Complex	Solvent	H_a_	H_b_	H_f_	H_c_	H_e_	H_d_	other
**1**	D_2_O	-	-	5.53	3.23	2.81	2.51	-
**1**	DMSO-d_6_	7.79	6.91	5.40	2.80	2.49	2.37	0.74(OH)
**2**	D_2_O	-	-	5.52	3.31	2.81	2.50	2.08(CH_3_)
**2**	DMSO-d_6_	8.53	8.33	5.43	2.92	2.61	2.34	1.95(CH_3_)
**3**	Acetone-d_6_	9.08	8.29	5.55	3.55	2.99	2.65	7.92(CHg) 7.55(CHh) 7,43(CHi)
**3**	DMSO-d_6_	8.69	8.32	5.47	3.13	2.68	-	7.87(CHg) 7.56(CHh) 7.45(CHi)
**4**	Acetone-d_6_	7.75	6.98	5.59	3.47	2.97	2.13	-
**4**	DMSO-d_6_	8.40	7.69	5.39	2.87	2.55	-	-
**5**	CD_3_OD	-	-	5.49	3.17(c) 3.11(c’)	2.71	2.49(d) 2.39(d’)	2.06(CH_3_)
**5**	DMSO-d_6_	8.52	7.76	5.41	2.89	2.59	2.13	-
**6**	CD_3_OD	-	-	5.48	3.13(c) 3.07(c’)	2.68	2.48(d) 2.42(d’)	-
**6**	DMSO-d_6_	8.11(a) 7.27(a’)	7.89(b) 7.27(b’)	5.40	3.04	2.84	2.40	1.03(OH)

**Table 2:** Selected ^13^C NMR chemical shifts (ppm) for compounds **1**–**6**.

Complex	Solvent	OC(O) oxa	C_f_	C_c_	C_d_/C_e_	Other
**1**	DMSO-d_6_	164.7	124.3	57.1	30.9	-
**2**	DMSO-d_6_	163.3	124.3	57.8	31.5	178.3(OC(O)) 22.9(CH_3_)
**3**	Acetone-d_6_	163.6	129.1	60.1	33.4	133.4(Cg) 133.3(Ch) 130.6(Ci)
**4**	Acetone-d_6_	163.4	125.0	60.0	32.8	-
**5**	CD_3_OD	166.7	125.4(Cf) 125.2(Cf’)	60.3(Cc) 60.2(Cc’)	33.1(Cd) 32.9(Cd’)	23.9(CH_3_)
**6**	CD_3_OD	167.1	125.4(Cf) 125.2(Cf’)	60.2(Cc) 59.3(Cc’)	33.1(Cd) 32.6(Cd’)	-

**Table 3:** Cathodic reduction potentials (E_p_^c^, Volt, values normalized vs. SCE) for the complexes **1**–**6** and log*P*_o/w_ (determined by the shake-flask method).

Complex	Potential (V)	log*P*o/w
**1**	−1.05	−3.05
**2**	−1.03	−2.69
**3**	−0.99	−0.26
**4**	−0.72	−1.65
**5**	−0.82	−2.03
**6**	−0.48	−2.39
**[Pt(OXA)(DACHEX)]**	−	−2.43

**Table 4:** Relative abundance of the two Pt oxidation states as derived from XPS analysis at the onset of the experiment and after 30 min irradiation and % of reduction for compounds **1**–**6**. % of reduction was calculated as Pt(IV)_30 min_/(Pt(IV)_0_ + Pt(IV)_30 min_).

% Relative Abundance Pt(IV)/(II)			
	As Irradiated	After 30 min Irradiation	% of Reduction
**1**	86/14	48/52	44
**3**	68/32	23/77	66
**2**	65/35	23/77	65
**4,5**	50/50	17/83	67
**6**	45/55	17/83	64

**Table 5:** Cytotoxic activity in vitro evaluated by MTT test at 72 h. IC_50_ values were calculated with a four-parameters logistic model (*p* < 0.05). CDDP: cisplatin, OXP: oxaliplatin.

Compound	IC_50_ (µM) ± SD					
	A431	2008	HCT-15	PSN-1	LoVo	LoVo OXP
**1**	0.49 ± 0.2	0.72 ± 0.1	0.44 ± 0.1	3.15 ± 0.8	0.84 ± 0.3	>2
**2**	0.55 ± 0.1	0.33 ± 0.5	1.27 ± 0.5	2.85 ± 0.7	0.59 ± 0.1	>2
**3**	0.16 ± 0.07	0.14 ± 0.03	0.41 ± 0.1	0.54 ± 0.3	0.07 ± 0.02	0.09 ± 0.02 (1.3)
**4**	0.43 ± 0.2	0.42 ± 0.1	1.17 ± 0.2	3.08 ± 1.1	0.39 ± 0.03	>2
**5**	1.51 ± 0.6	0.93 ± 0.3	1.97 ± 0.4	3.58 ± 1.4	0.25 ± 0.05	>2
**6**	1.31 ± 0.7	1.87 ± 0.4	1.67 ± 0.4	5.25 ± 1.2	0.66 ± 0.02	>2
**CDDP**	1.43 ± 0.4	2.23 ± 0.1	15.28 ± 2.6	16.6 ± 4.2	7.6 ± 1.5	-
**OXP**	3.8 ± 1.3	1.53 ± 0.9	1.15 ± 0.4	7.9 ± 3.5	1.1 ± 0.6	14.3 ± 2.1 (13)

**Table 6:** Cytotoxicity towards human A431 cancer cell spheroids. Spheroids were treated for 72 h with increasing concentrations of tested compounds. The growth inhibitory effect was evaluated by APH. IC_50_ values were calculated by 4-parameters logistic model (*p* < 0.05). SD = standard deviation. CDDP: cisplatin, OXP: oxaliplatin.

		IC_50_ (µM) ± SD						
	1	2	3	4	5	6	CDDP	OXP
**A431**	5.8 ± 0.8	3.4 ± 0.9	1.8 ± 0.4	9.9 ± 2.6	26.4 ± 5.5	30.2 ± 3.8	58.3 ± 0.9	36.3 ± 1.2

## Supplementary Materials

Supplementary materials can be found at https://www.mdpi.com/1422-0067/21/7/2325/s1.

## Abbreviations

APH	Acid phosphatase
BzO	Benzoate
CDDP	Cisplatin
CV	Cyclic voltammetry
DACH	Diaminocyclohexane
DACHEX	Diaminocyclohexene
HSQC	Heteronuclear single quantum spectroscopy
ICP-AES	Inductively coupled plasma atomic emission spectroscopy
MTT	3-(4,5-dimethylthiazol-2-yl)-2,5-diphenyltertrazolium
OXP	Oxaliplatin
SCE	Saturated calomel electrode
SDS	Sodium dodecyl sulfate
XPS	X-ray photoelectron spectroscopy
