# Supplementary material for: Platinum(IV) Complexes of trans-1,2-diamino-4-cyclohexene: Prodrugs Affording an Oxaliplatin Analogue that Overcomes Cancer Resistance
Source: Int J Mol Sci. 2020 Mar 27;21(7):2325. doi: 10.3390/ijms21072325 (PMC7177638; doi:10.3390/ijms21072325)
Supplement: Supplementary file 1 [file ijms-21-02325-s001.pdf]

Platinum(IV) complexes of *trans*-1,2-diamino-4-cyclohexene: prodrugs affording an oxaliplatin analogue that overcomes cancer resistance.

Paride Papadia,[a] Katia Micoli,[b] Alessandra Barbanente,[b] Nicoletta Ditaranto,[b] James D. Hoeschele,[c] Giovanni Natile,[b] Cristina Marzano,[d] Valentina Gandin,[d] Nicola Margiotta[b,*]

[a]*Department of Biological and Environmental Sciences and Technologies (DiSTeBA), University of Salento, 73100 Lecce, Italy;*

[b]*Dipartimento di Chimica, Università degli Studi di Bari Aldo Moro, Via E. Orabona 4, 70125 Bari, Italy;*

[c] *Department of Chemistry, Eastern Michigan University, 48197 Ypsilanti, MI (USA);*

[d] *Dipartimento di Scienze del Farmaco, Università di Padova, Via Marzolo 5, 35131, Padova, Italy.*

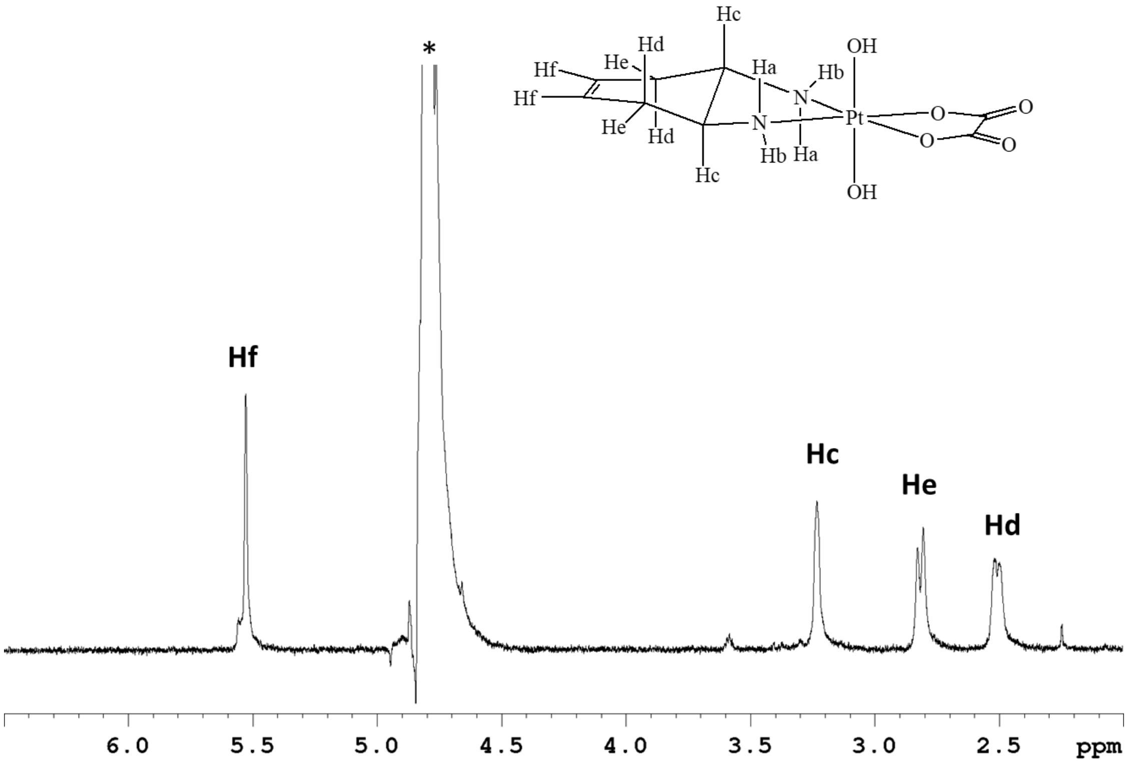

Figure S1. *^{1}H-NMR spectrum (700 MHz, ^{1}H) of **1** in D_2O. * marks solvent residual peaks.*

The amino proton signals are not visible due to the rapid exchange with D_2O. The singlet falling at 5.53 ppm was attributed to vinyl H_f protons while the signals relating to methynic H_c protons resonate to lower fields (3.23 ppm) compared to DMSO-d_6 (2.80 ppm). Methylenic protons He resonate at 2.81 ppm and H_d protons at 2.51 ppm. The deshielding of these signals could be attributed to the improved solvation properties of water molecules relative to DMSO.

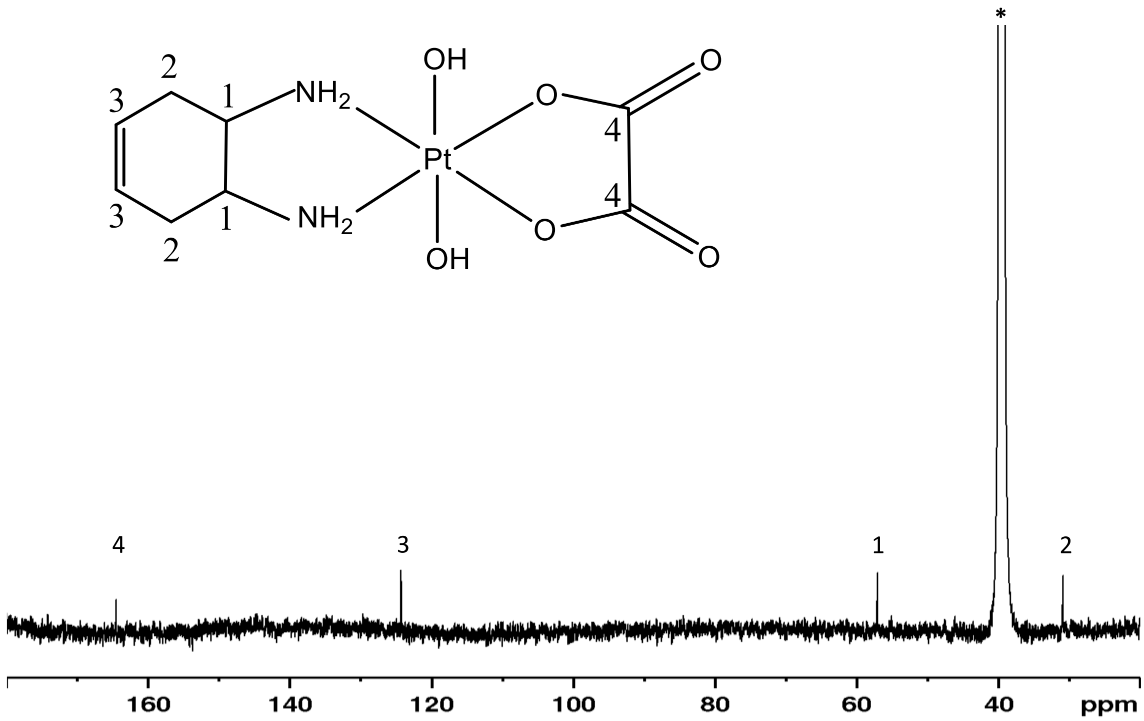

Figure S2. *13C-NMR spectrum (176.05 MHz, 13C) of* ***1*** *in* DMSO-d6. ** marks solvent residual peaks.*

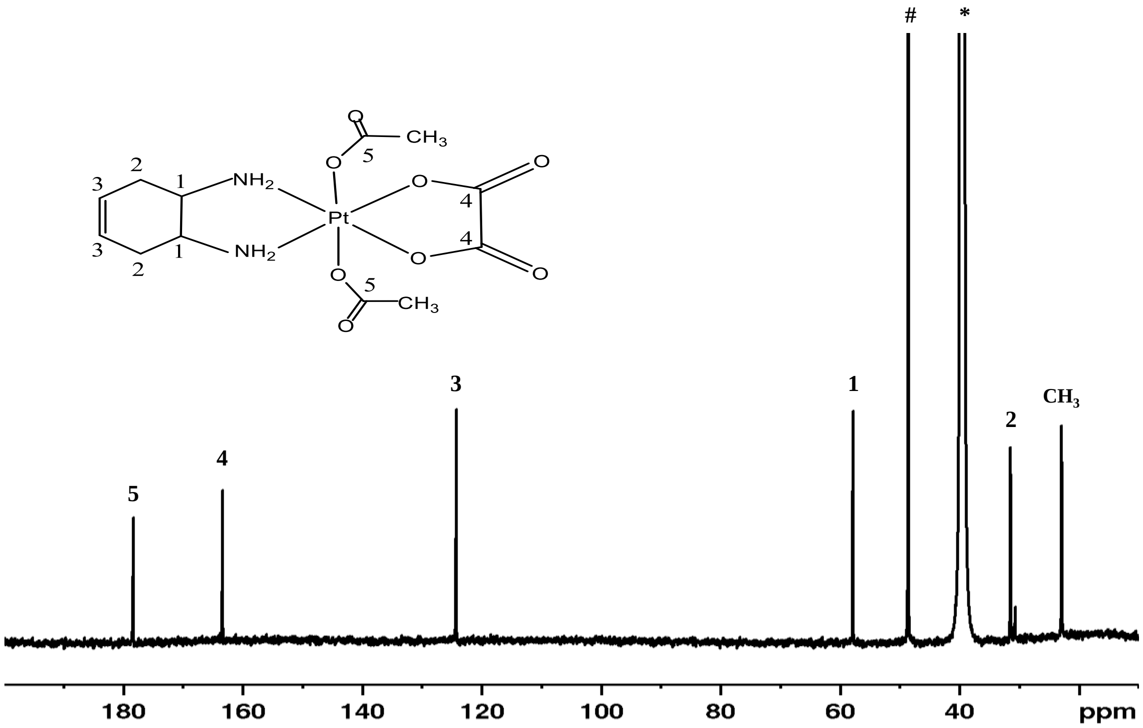

Figure S3. *^{13}C-NMR (176.05 MHz, ^{13}C) spectrum of* ***2*** *in DMSO-d$_6$. * marks residual solvent peak. # marks an impurity (methanol) in the sample*

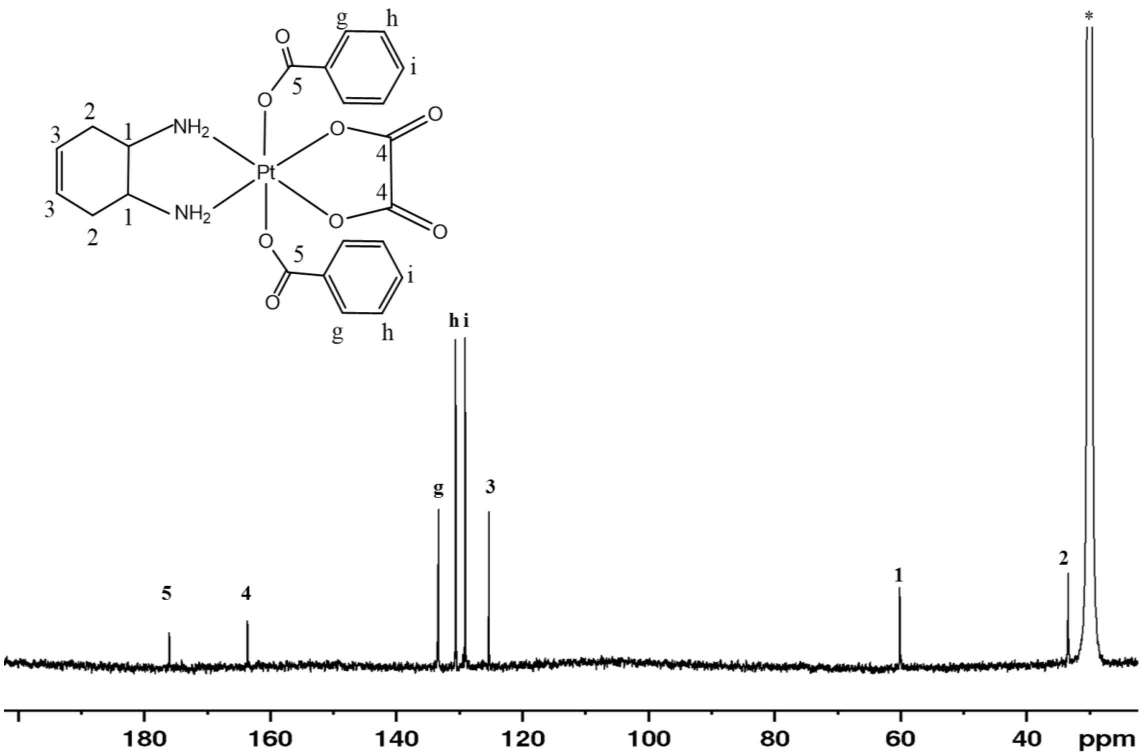

Figure S4. *^{13}C-NMR (176.05 MHz, ^{13}C) spectrum of **3** in acetone-d_6. * marks residual solvent peak.*

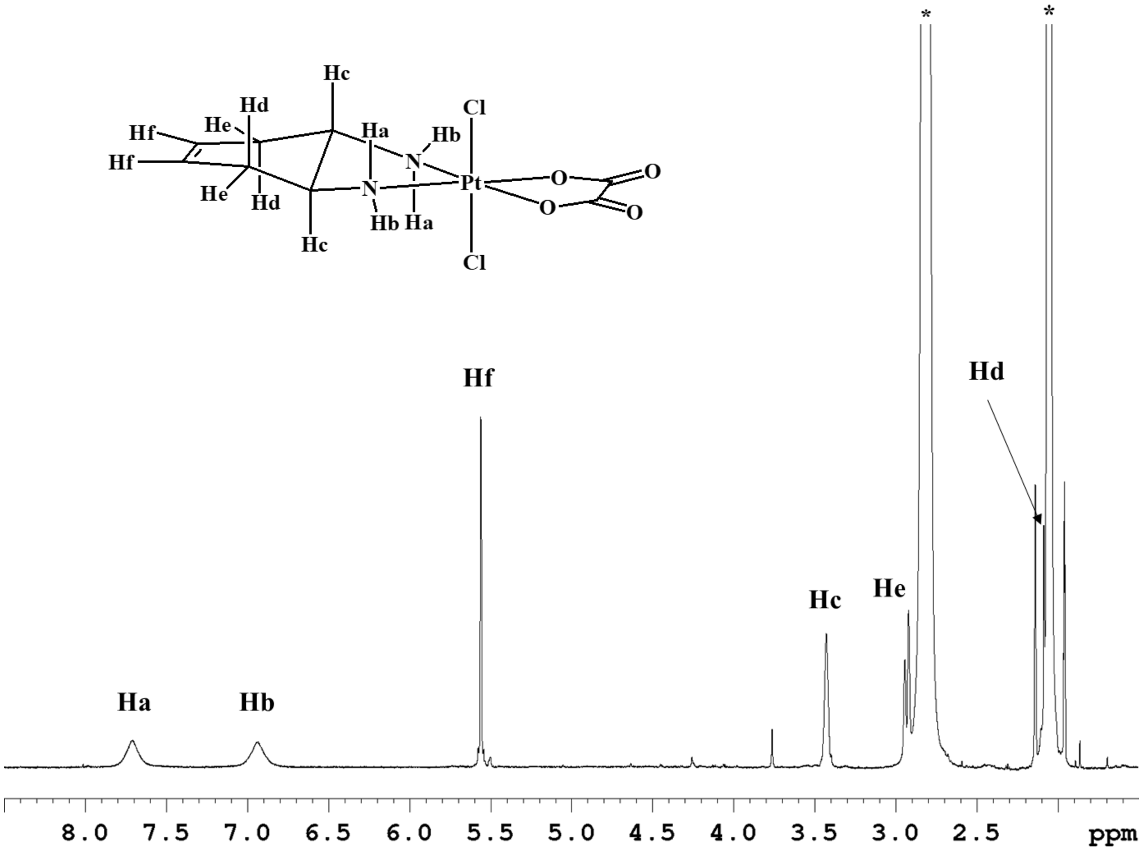

Figure S5. *^{1}H-NMR spectrum (700 MHz, ^{1}H) of* ***4*** *in Acetone-d_6. * mark solvent residual peaks.*

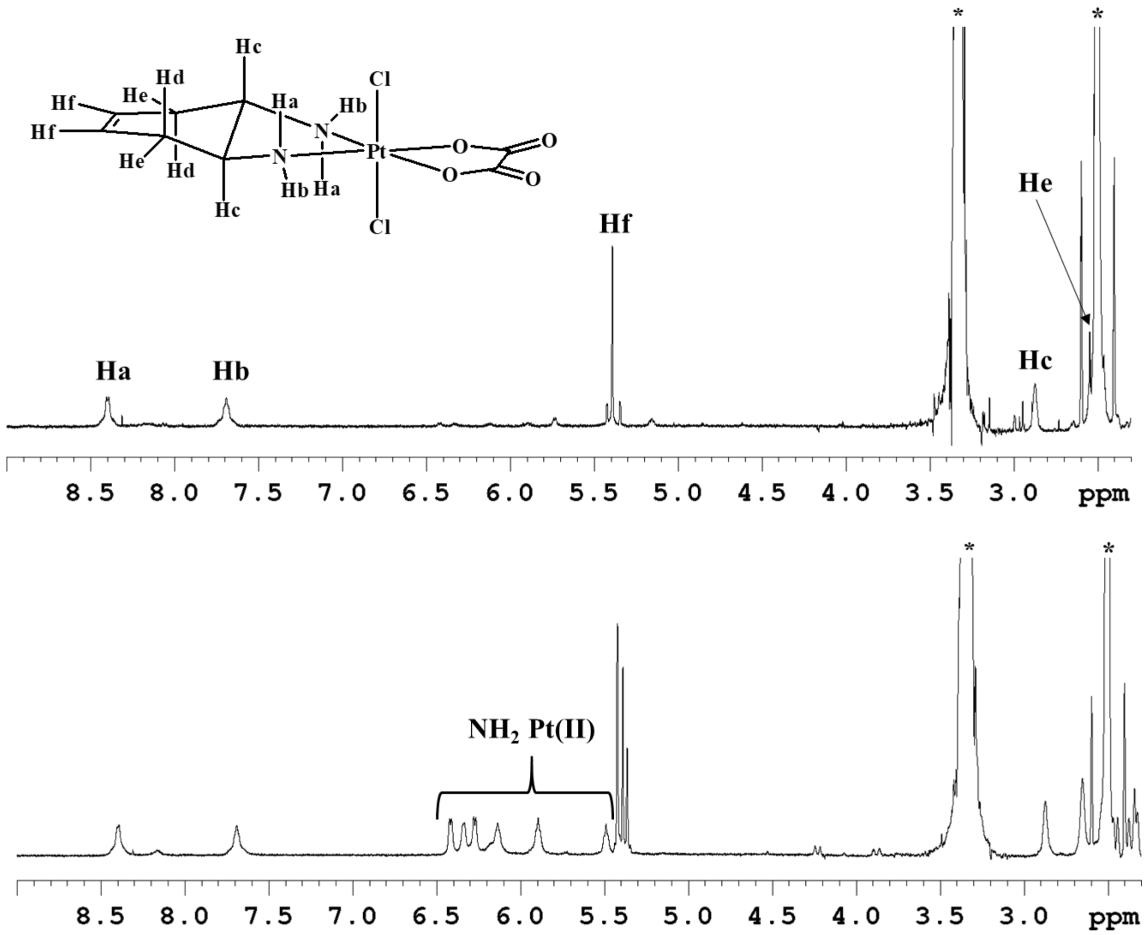

Figure S6. *^{1}H-NMR spectra (700 MHz, ^{1}H) of **4** in DMSO-d_6 recorded after 10 minutes (top) and 1 day (bottom). * marks solvent residual peaks.*

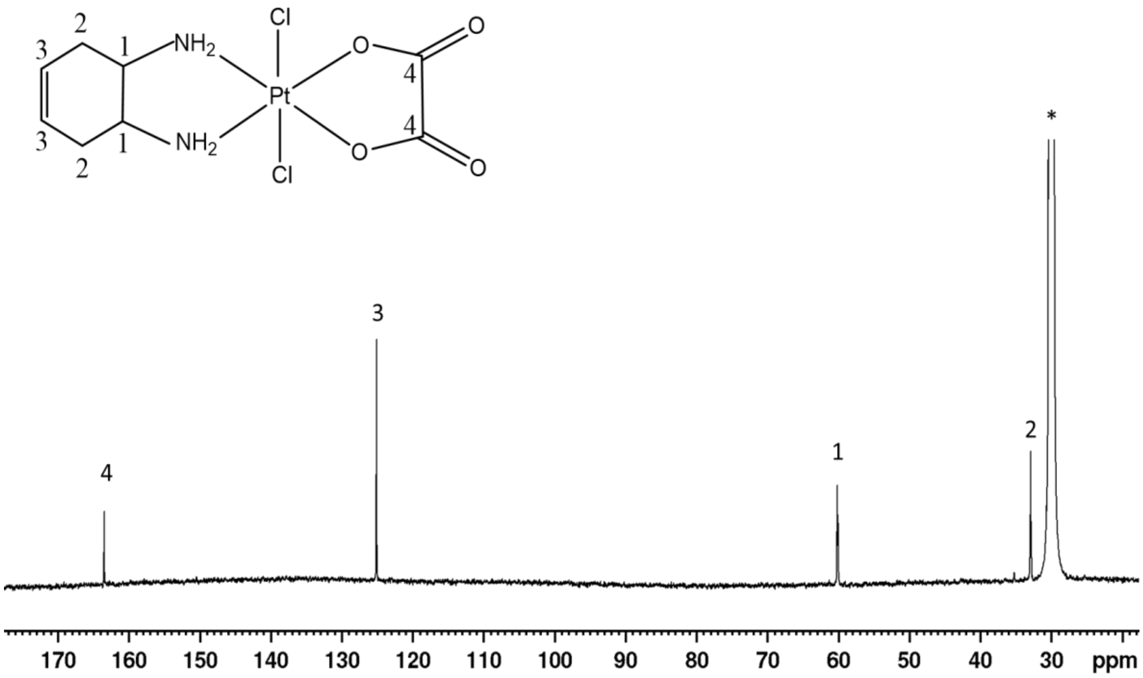

Figure S7. *^{13}C-NMR (176.05 MHz, ^{13}C) spectrum of* ***4*** *in acetone-d_6. * marks residual solvent peak.*

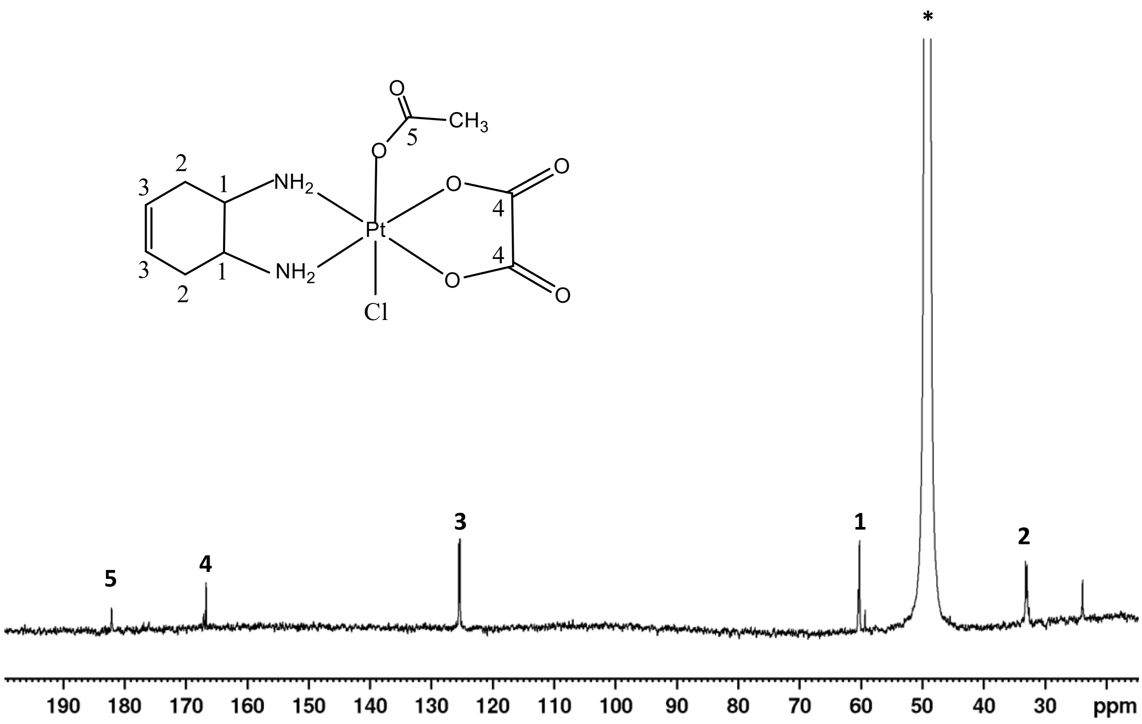

Figure S8. *^{13}C-NMR (176.05 MHz, ^{13}C) spectrum of **5** in CD_3OD. * mark residual solvent peaks.*

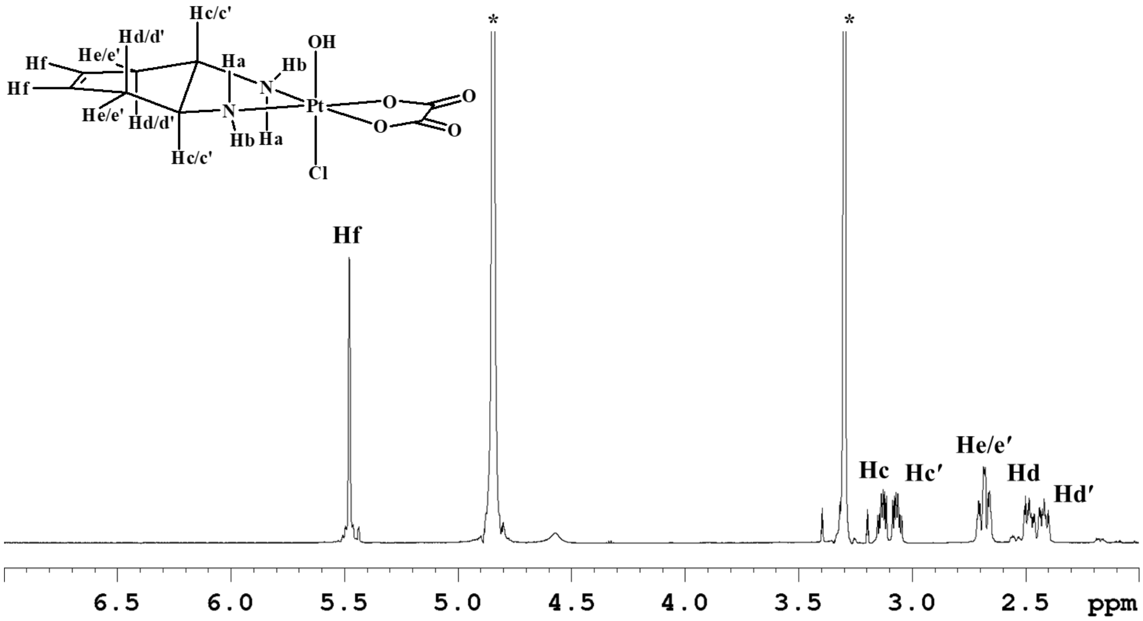

Figure S9. *^{1}H-NMR (700 MHz, ^{1}H) spectrum of complex **6** in CD_3OD. * mark residual solvent peaks.*

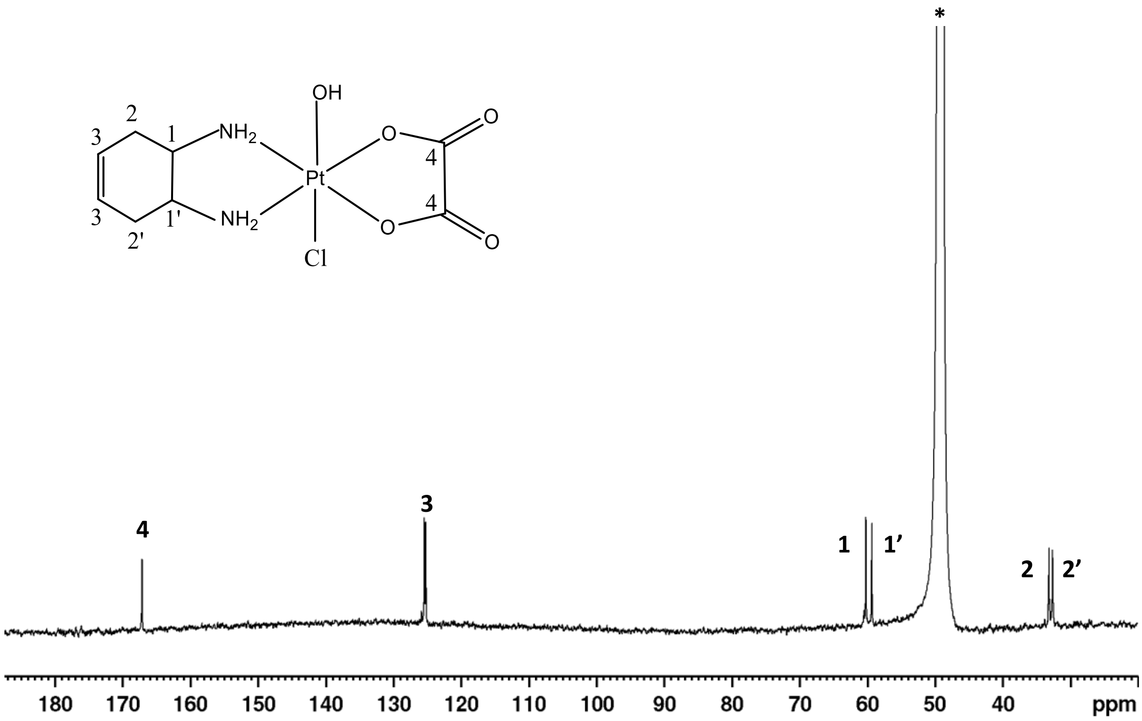

Figure S10. *^{13}C-NMR (176.05 MHz, ^{13}C) spectrum of* ***6*** *in CD_3OD. * mark residual solvent peaks.*

Table S1. Cathodic reduction potential for complexes **1-6**, with the corresponding ligand total electronegativity $\chi_{A(T)}$, and relative averages.

**Complex**	**Axial ligand 1**	**Axial ligand 2**	**Ep^c (V)**	**$\chi_{A(T)}{}^1$**	**$\chi_{A(T)}{}^2$**	**average $\chi_{A(T)}$**
**1**	OH	OH	-1.05	3.99	3.99	3.99
**2**	AcO	AcO	-1.03	4.04	4.04	4.04
**3**	BzO	BzO	-0.99	4.06	4.06	4.06
**4**	Cl	Cl	-0.72	4.23	4.23	4.23
**5**	AcO	Cl	-0.82	4.04	4.23	4.14
**6**	OH	Cl	-0.48	3.99	4.23	4.11

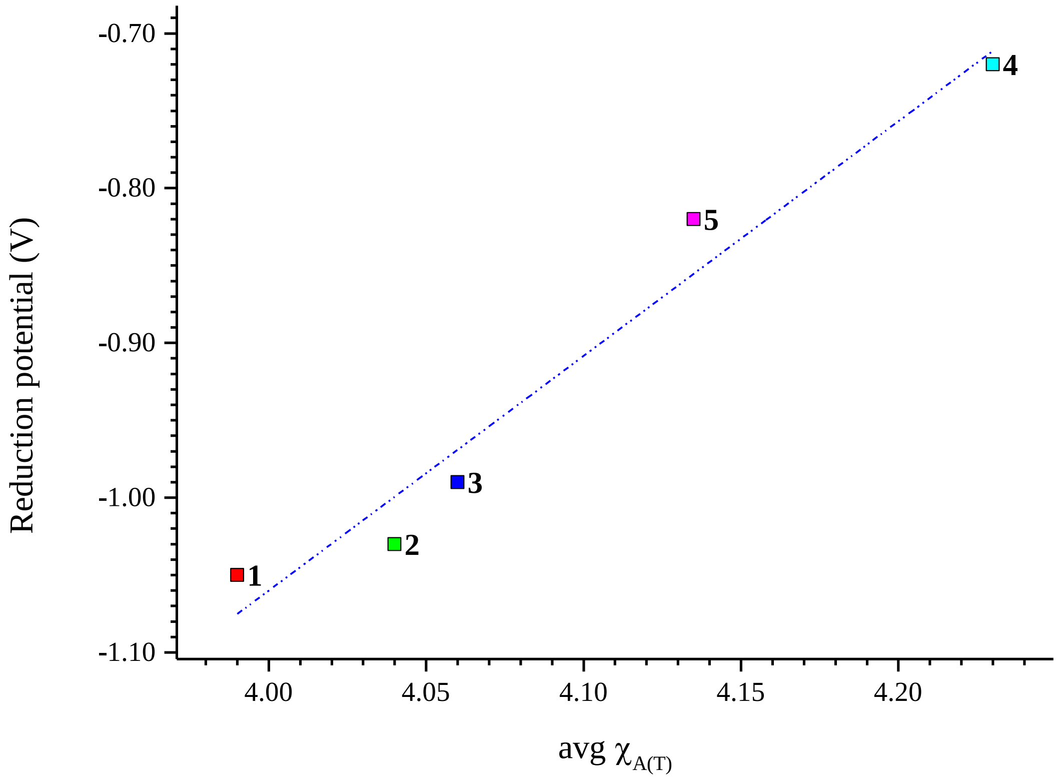

Figure S11. Scatterplot of the average $\chi_{A(T)}$ values vs. the cathodic reduction potential for the complexes **1-5**. The dashed line represents the best linear fit: Ep^c (V) = -7.13 +1.52·$\chi_{\mathbf{A(T)}}$ (Adjusted R^2 = 0.94698, p-value < 0.01).

a b

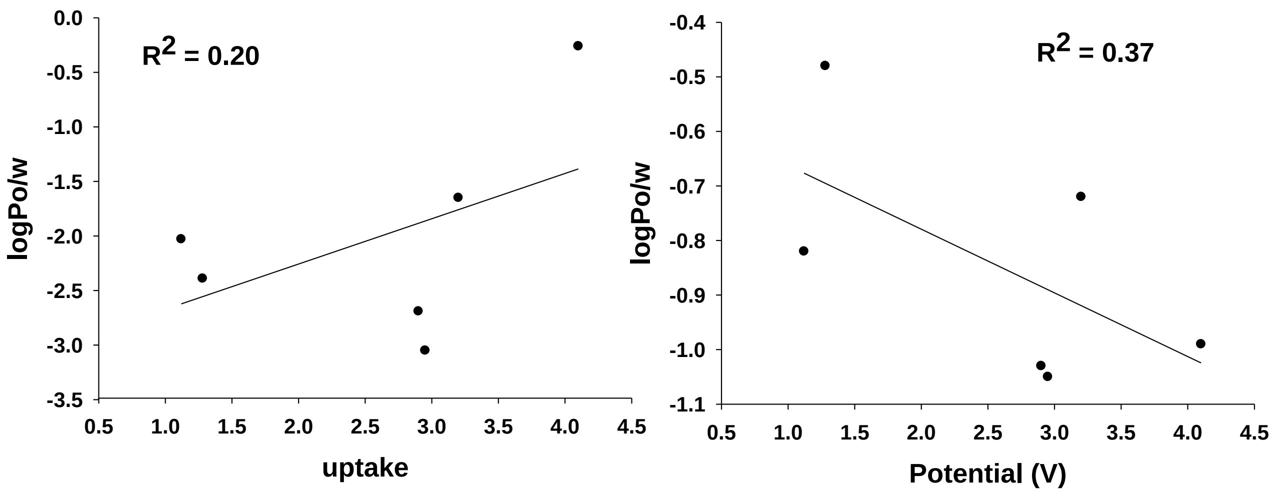

Figure S12. Correlation between log$P_{o/w}$ and cellular uptake (a) and reduction potential (b)
